# Family functioning and eating disorders in Chinese populations: a systematic review and meta-synthesis

**DOI:** 10.1186/s40337-025-01453-1

**Published:** 2025-11-24

**Authors:** Xu Han, Mei-chun Cheung, Jacqueline Corcoran

**Affiliations:** 1 Department of Social Work, The Chinese University of Hong Kong, Shatin, New Territories, Hong Kong SAR, China; 2 School of Social Policy and Practice, University of Pennsylvania, Philadelphia, USA

**Keywords:** Family functioning, Eating disorders, Cultural factors, Chinese

## Abstract

**Objective:**

Familial factors play crucial roles in the development of eating disorders and related psychopathology. However, the generalizability of findings from Western studies on familial influences to Chinese populations remains questionable. This review systematically examines the relationship between family functioning, conceptualized within the family process model, and the development of eating disorders in Chinese patients.

**Methods:**

Empirical studies were systematically reviewed on the basis of family process theory. The quantitative data were integrated and synthesized with qualitative data using a convergent integrated approach. A thematic analysis was conducted using NVivo 12. This review adhered to the Preferred Reporting Items for Systematic reviews and Meta-Analyses 2020 Checklist and was registered with PROSPERO.

**Results:**

Fifty studies (13 quantitative and 37 qualitative or case studies) highlighted six themes: (1) family values; (2) family tasks: individuality; (3) family communication: conflict avoidance and affective involvement; (4) parental roles; (5) parental control; and (6) specific behaviours (e.g., unhealthy feeding practices and negative family body talk). These factors may play a role in the development of eating disorders and provide insight into the dynamics within Chinese families.

**Conclusion:**

Filial piety and gender preferences characterize cultural contexts in patients’, families’, and professionals’ narratives. While individuality and eating-related behaviours appear in both Chinese and Western contexts, notable differences exist. Avoidant family communication, emotional disengagement, parental control, and strong academic focus were identified, but parental roles and divisions showed no consistent patterns. Quantitative findings only partially support the qualitative descriptions.

**Supplementary Information:**

The online version contains supplementary material available at 10.1186/s40337-025-01453-1.

## Abstract (plain language summary)

This review explored how family relationships and dynamics might contribute to eating disorders specifically within Chinese populations. Because most existing research on family influences comes from other cultural contexts, the researchers focused on understanding the unique aspects relevant to Chinese families. They systematically analysed 50 existing studies involving Chinese patients, using a model that examines how families interact and function.

The analysis identified several key family themes linked to eating disorders in this group. These include the strong influence of cultural values like respect for parents (filial piety) and gender preferences, different individuality within the family, parental roles and divisions, communication patterns that avoid conflict, low emotional connection, high levels of parental control, an intense focus on academic achievement, and specific behaviours like unhealthy feeding practices or negative talk about body image at home. However, the evidence from in-depth interview studies was only partially matched by findings from statistical studies, indicating a need for further research to solidify understanding of these specific family dynamics and their role in eating disorders among Chinese individuals.

**Supplementary Information:**

The online version contains supplementary material available at 10.1186/s40337-025-01453-1.

## Introduction

Eating disorders are characterized by persistent disturbances in eating behaviours or related activities, leading to significant alterations in food consumption or absorption. These disturbances can severely impact both physical health and psychosocial functioning [1]. The three primary types of eating disorders are anorexia nervosa, bulimia nervosa, and binge eating disorder. The key behavioural manifestations of these disorders include disordered eating patterns, excessive dieting, episodes of binge eating, and purging behaviours [2], all of which constitute the core pathology of eating disorders. Eating disorders begin in adolescence or young adulthood [1, 3], between 15 and 25 years in girls and 10 and 14 years in boys [3], and the age of onset has been decreasing [4]. In 2019, 14 million people had eating disorders, including approximately 3,000,000 children and adolescents [5]. Biological (e.g., genetic predisposition), psychological (e.g., personality, emotions, and cognitive traits), and social (e.g., peer and media influences) factors significantly contribute to the development of eating disorders [6]. As eating disorders predominantly begin in adolescence [1, 3], family factors, although not causative, may be involved [7].

Family functioning is frequently referenced when discussing the familial factors associated with eating disorders [8]. According to the family process model [9], family functioning is a dynamic operational system encompassing values, task completion, roles, communication, control, and behaviours [9, 10]. Values reflect cultural and societal influences that shape how family members interact, the roles they assume, and the tasks they prioritize. Within families, task accomplishment involves collaborative efforts to achieve shared goals, with distinct developmental objectives at various stages of the family life cycle. Role performance pertains to the effectiveness with which individual members fulfil the expected behaviours, responsibilities, and functions associated with their family roles. Communication encompasses the exchange of information, which encompasses affective expression as well as instrumental and neutral content. Control describes the influence that family members exert on one another. Behaviour serves as the external manifestation of all the elements. Together, these elements create a comprehensive framework for understanding family functioning, illustrating how they interrelate and affect individual development [9].

Western researchers have extensively investigated family functioning and its dimensions in relation to eating disorders [11]. A comprehensive review (The search terms used are provided in Supplementary Material A) identified seven English-language systematic or scoping reviews [12–18] that synthesized evidence from 109 studies among samples from Western countries. These reviews collectively examined family factors associated with eating disorders, with a focus on parental appearance-related teasing [13], family mealtime dynamics [12], adverse experiences [14], parenting styles [15], and family pressures [17]. The included studies were predominantly observational (99.08%), with 96.33% employing quantitative, mainly cross-sectional designs (76.15%). Most studies focused on adolescents aged 10–19 years, whereas some [12, 14] employed retrospective methods to examine family influences during childhood and adolescence without age restrictions. Both clinical and community samples are represented. None of the reviews conducted a meta-synthesis, and only one review performed a meta-analysis [17]. The detailed findings are summarized in Table 1 and Supplementary Material A.

**Table 1:** Summary of articles included in Western systematic reviews

Variable		Option	*N*	Valid percentage
Gender		Mixed gender	68	62.39%
		Only female	39	35.78%
		Only male	2	1.83%
Methodology	Observational vs. Experimental	Observational	108	99.08%
		Experimental	1	0.92%
	Quantitative vs. Qualitative vs. Mixed methods	Quantitative	105	96.33%
		Qualitative	2	1.83%
		Mixed methods	1	0.92%
	Cross sectional vs. Longitudinal	Cross sectional	83	76.15%
		Longitudinal	25	22.94%
Sample site		Australia	15	13.76%
		Austria	1	0.92%
		Belgium	5	4.49%
		Canada	4	3.67%
		Finland	1	0.92%
		France	1	0.92%
		Germany	9	8.26%
		Italy	4	3.67%
		Netherlands	4	3.67%
		Portugal	4	3.67%
		Spain	1	0.92%
		Sweden	1	0.92%
		Switzerland	1	0.92%
		UK	15	13.76%
		USA	42	38.53%
		Cannot tell (due to inability to access the full text)	1	0.92%

When viewing previous reviews through the lens of the family process model, general family functioning was addressed in two reviews [16, 18]. Communication and affective involvement were highlighted by some reviews [12, 14, 16]. Control and behaviours related to family functioning were discussed in the reviews by Grogan, MacGarry [14], Hampshire, Mahoney [15], Dahill, Touyz [13], Godfrey et al. [12], Langdon-Daly and Serpell [16], and Quiles Marcos, Quiles Sebastián [17].

In general, supportive family environments and positive familial relationships significantly reduce the risk of eating disorders among adolescents [16]. Conversely, other familial factors, such as parental weight, education level, and socioeconomic status, are not significantly associated with pathological eating behaviours in children [18].

In terms of communication and affective involvement, family support and connectedness can promote general adaptive development and a range of positive outcomes, although these outcomes are not specific to eating disorders [16]. Conversely, insufficient intimacy, family disharmony, and low family involvement and support among family members may serve as risk factors [14, 16]. Godfrey, Rhodes [12] also emphasized the significant positive association between mealtime conflict and children’s disordered eating behaviours.

For control and behaviours related to family functioning, negative parenting styles characterized by high levels of control and low levels of responsiveness are associated with a heightened likelihood of developing eating issues [14, 15]. Feeding practices, comments, and concerns regarding a child’s weight, and critical attitudes towards body shape and appearance can act as risk factors for the development of eating disorders [12, 13, 16, 17].

However, the results have limited applicability since the studies involve samples from Western countries, which typically feature nuclear family structures, encourage independence, and emphasize open emotional expression [19]. In contrast, Chinese family culture is characterized by collectivism, filial piety, and hierarchical relationships, emphasizing the importance of family unity and respect for elders [20]. Chinese immigrants residing in the United Kingdom tend to uphold traditional Chinese parenting values, emphasizing obedience and self-discipline, as well as filial piety. Additionally, such parents encourage modesty, humility, and consideration for others, aiming to cultivate social harmony and interpersonal sensitivity within their children [21]. In the context of eating pathology, the review by Sun, Soh [22] primarily draws upon cross-sectional studies, which indicate that among Asian students in the United States and the United Kingdom, intergenerational conflict, maternal overprotection, and achievement-oriented family dynamics are significantly elevated compared with their non-Asian peers, and these familial stressors are associated with increased levels of eating pathology. However, further exploration of Chinese family functioning among individuals with eating disorders warrants scholarly investigation. Given the distinctions between Chinese and Western contexts, a thorough review and analysis of family functioning and eating disorders within the Chinese population is essential.

## Current study

In the present study, we aim to elucidate the various aspects of family functioning associated with the development of eating disorders in Chinese individuals on the basis of the family process model.

## Methodology

### Search method

This systematic review was conducted from 2023 to 2024 and followed the Preferred Reporting Items for Systematic reviews and Meta-Analyses (PRISMA) 2020 Checklist (Supplementary Material I). In the literature search, we adhered to the following criteria: literature published between January 1990 and December 2023 from PubMed, EBSCO host databases (including PsycINFO, APA PsycINFO, APA PsycArticles, Medline, Psychology, and Behavioural Sciences), SCOPUS, Web of Science and the Chinese Social Sciences Citation Index (CSSCI) was included. In addition to the primary database searches, supplementary searches were conducted using Google Scholar and the university library to identify additional relevant literature that was not captured through the initial search strategy. These supplementary searches involved manual screening and retrieval of pertinent articles that emerged during the review process. The specific search terms and data are detailed in Supplementary Material B.

#### Definitions of keywords

*Eating disorders* include clinical eating disorders diagnosed by standard criteria, as well as disordered eating behaviours and symptoms measured in community samples [23]. It does not include obesity, intuitive eating, or body dissatisfaction.

*Family functioning* is defined according to the family process model, and includes family task accomplishment, communication, role performance, affective involvement, control, and values and norms [9].

#### Keywords related to eating disorders

“eating disorder*” OR “anorexia” OR “bulimia” OR “binge eat*” OR “eating symptom*” OR “eating disturbance” OR “disordered eating” in the article title, abstract, and/or keywords [24, 25].

#### Keywords related to the location

Chin*” OR “Hong Kong” OR “Taiwan*.

#### Keywords related to family

famil*.

### Eligibility criteria

The population, phenomenon of interest, context, and study design (PICoS) strategy was used to define the eligibility criteria.

#### Population

##### Inclusion

1. Clinical samples (adolescents/adults with diagnosed anorexia nervosa, bulimia nervosa and binge eating disorder).
2. Community samples (individuals exhibiting disordered eating behaviours across both age groups).

##### Exclusion

1. Studies that include a multi-ethnic sample, but did not distinguish Chinese participants from other groups in the main analyses (e.g., Shagar, Donovan [26] and Lee and Lock [27]).
2. Studies on avoidant/restrictive food intake disorders were excluded because of the distinct psychopathological mechanisms and clinical presentation of the disorder compared with anorexia nervosa, bulimia nervosa, and binge eating disorder and the associated complex familial dynamics [28].

#### Phenomenon

##### Inclusion

1. Studies addressing factors associated with the development of eating disorders.
2. Studies addressing the treatment of eating disorders with case formulation and conceptualization.

##### Exclusion

1. Studies that focused solely on subjective experiences related to treatment, such as Sun, Lam [29].
2. Studies that focused solely on postonset dynamics (e.g., Zhang, Wu [30]).

#### Context

##### Inclusion

Studies that focused on the context of Chinese families.

##### Exclusion

Studies that addressed only peer or media influence.

#### Study design

##### Inclusion

Academic empirical qualitative or quantitative studies. Case studies focusing on one or a few cases were also included in this study.

##### Exclusion

Reviews, scale translations, and theoretical pieces.

### Data extraction

Two reviewers (Authors 1 and 2) screened titles and abstracts to assess their relevance, followed by review of the full texts and references. Studies were selected on the basis of the eligibility criteria, and differences were resolved through consensus. Excel and EndNote were used for documentation.

### Data collection

In the data collection process, Author 1 initially extracted relevant data from the included reports, focusing on participant characteristics, study methods, and key themes. An assistant independently reviewed the extracted data to ensure accuracy and consistency. Excel was used to record and organize the extracted data.

### Quality appraisal

The Quality Assessment Tool for Observational Cohort and Cross-Sectional Studies [31] was used to evaluate the risk of bias and overall research quality. The assessment tool includes 14 criteria, including design, selection bias, data collection, and confounders. Considering that items 6, 7, 10, and 13 are primarily designed to assess the quality of longitudinal studies, and given that none of the included studies employed a longitudinal design, these items were not evaluated in the present review. The Critical Appraisal Skills Program Qualitative Checklist [32], comprising 10 criteria, including study design, data collection, and the credibility and generalizability of the findings, was used to evaluate qualitative studies. Given their inherently descriptive and exploratory nature that aligns with qualitative research methodologies, case studies were also appraised using this checklist. Two researchers assessed the quality as “good,” “fair,” or “poor.” Differences in ratings were discussed with a third researcher to reach consensus. The specific criteria for the evaluation are detailed in Supplementary Material F & G.

### Data synthesis strategy

*Quantitative Data Processing* The quantitative data were synthesized using a convergent integrated approach, as demonstrated in mixed-methods reviews [33, 34]. Numerical data underwent “qualitizing,” a process in which quantitative findings were translated into textual descriptions and themes to facilitate integration with qualitative data [35]. Initial coding was performed to identify key patterns, followed by iterative refinement to develop descriptive and analytical themes.

*Qualitative Data Processing* In this study, Thomas and Harden’s thematic synthesis approach for qualitative data [36] was employed to systematically integrate findings from multiple qualitative data sources. The process involved three distinct stages [36]. The first stage entailed line-by-line coding of the primary qualitative texts, allowing for an in-depth and nuanced understanding of the data. The second stage focused on developing descriptive themes that remained closely aligned with the original studies, providing a transparent synthesis of the primary findings [36]. The final stage involved generating analytical themes that went beyond the descriptive level, and the family process model [9] was used as a theoretical framework to interpret and contextualize the findings [36].

The results and discussion sections concerning the interpretation and discussion of the findings were utilized for coding. All coding, thematic organization, and analytical theme development were conducted using NVivo 12 software. Themes and subthemes were iteratively reviewed and refined until a consensus was reached. Some articles reported that similar case scenarios and dialogues, such as Chan [37], Chan and Ma [38], Chan and Ma [39], and Chan and Ma [40], were not subjected to duplicate coding; however, their discussions were distinctly analysed.

## Results

### Search outcomes

**Fig. 1:**
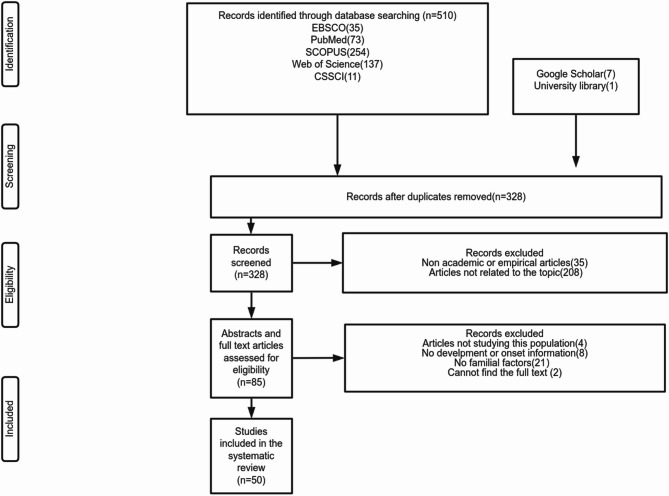
PRISMA flowchart depicting the study selection process

A total of 518 articles were retrieved, of which 50 (13 quantitative and 37 qualitative or case studies) were ultimately reviewed (Fig. 1). Among the included samples, twenty-one originated from mainland China, seventeen from Hong Kong, four from Taiwan, two each from France and the United Kingdom, and one each from the United States and Singapore. Additionally, one sample included both Taiwan and the United States, and another included both Hong Kong and mainland China. Two of the studies were doctoral dissertations, while the remaining were published journal articles. Four studies were reported in French, three in Chinese, and the remainder in English. The titles and publication years are shown in Supplementary Material C.

### Study characteristics

The characteristics and quality appraisal details are shown in Supplementary Materials D, E, F, and G. To ensure a comprehensive review, we deliberately retained all ratings and did not exclude any information.

### Meta-synthesis findings

Six main themes were identified.

*Theme 1: Family Values (n = 26)* In Chinese culture, filial piety and gender preferences are commonly observed family values.

1. *Filial Piety (n = 25)* Filial piety, a fundamental Confucian principle, connects parents and children and is associated with eating issues. It includes values such as obedience, gratitude, respect, and love for parents [41], emphasizing the children’s duty to mind their parents to obtain parental affection [37, 42]. It involves prioritizing parental wishes over personal desires, self-sacrificing for the family’s well-being, and fulfilling familial duties [41, 43–47].

Filial piety appears in the descriptions of both patients and family members; however, these accounts seem to be conflicting in nature. It emphasizes that one’s body belongs to one’s parents and that physical well-being is a hallmark of virtuous children [48–50]. Therefore, eating behaviours that maintain health express filial piety [51, 52]. Individuals who uphold filial piety should approach eating pathology with caution, as it may harm their health [49]. Some Chinese patients may exhibit atypical eating disorders, resist the desire to lose weight, and view weight loss as a selfish act [43] while emphasizing the importance of maintaining good health [48], which is consistent with the belief of filial piety.

Girls from low-income families in Hong Kong may restrict their food intake to fulfil family responsibilities rooted in filial piety and alleviate financial burdens [39, 42]. This dietary restriction may lead to thinness and eating-related problems [39, 42]. Filial piety holds that harming one’s body is akin to harming one’s parents [48, 49]; therefore, eating pathology can be difficult to tell parents due to guilt [49]. Eating pathology can thus become an indirect means of expressing discontent and asserting autonomy, as filial piety often prohibits the direct expression of disagreement [41, 43, 52, 53]. Food refusal and binge-purging behaviours are often seen as manifestations of the child’s internal conflict between loyalty to their parents and the desire for self-identity [47, 48, 54, 55]. A patient may conform to parental expectations and sacrifice for the family’s benefit before becoming sick [47, 56, 57]. However, after the onset of eating disorders, food refusal became a powerful weapon she used to fight for autonomy and to punish her parents [57].

In their quantitative study, Tsai, Curbow [58] acknowledged the potential influence of filial piety, which was addressed through relevant items included in the Taiwanese Ethnic Identity Scale. Although the results pertaining specifically to filial piety were not reported separately, it is evident that Taiwanese ethnic identity, encompassing filial piety-related items, demonstrated a significant positive association with disordered eating attitudes and behaviours.

2) *Gender preferences (n = 7)* The preference for sons over daughters in China is clear [37, 41, 44, 46, 47, 54, 59] and is discussed in many patients’ stories. Daughters are often expected to assume the responsibility of caring for family members and to demonstrate loyalty and filial piety towards their parents (Chan, 2003). For female patients, the perception that their brothers are favoured in the family can lead to feelings of neglect [37]. Food refusal is conceptualized as a manifestation of seeking parental attention and recognition [37]. In the case of a Chinese family described by Lee, Hsu [59], this dynamic was evident through the clear favouritism shown towards the patient’s two brothers. The patient’s father was emotionally distant, which contributed to the development of a dependent personality characterized by low self-esteem and ambivalence towards the patient’s domineering mother [59].

In another case, parents without a son experienced a lasting sense of shame within their extended family and community [47]. Consequently, the parents in this case projected their dissatisfaction and distress onto their daughter [47], who bore the full weight of their disappointment, as they were expected to compensate for the absence of a male child by assuming additional responsibilities and meeting heightened demands, which is the background of talking about eating disorders [47].

Another prevailing belief pertains to gender preferences: daughters are traditionally expected to leave their parental homes to reside with and become part of their husbands’ families [41, 46], whereas sons hold greater significance and are valued [37, 41, 44]. In the case presented by Ma [46], the patient’s parents expressed the view that “We old folks believe that a married daughter is like water being poured out of the front door” and perceived divorce as inevitably associated with sorrow. They also considered their daughter’s ex-husband to be a virtuous individual and rationalized his use of violence as a response to her bingeing and purging behaviours [46]. Their disapproval was indicative of the internalization of fundamentally ingrained cultural beliefs and normative standards pertaining to gender roles, marital expectations, and family responsibilities, without acknowledgement of the daughter’s personal suffering [46].

*Theme 2: Family Tasks: Individuality (n = 22)* Individuality is a crucial family developmental task concerning a child’s pursuit of autonomy, self-governance, and self-determination [45, 46, 60]. Ma [46] emphasized that the pursuit of individuality is not exclusive to Chinese patients. Studies posit that distorted eating patterns, to a certain extent, symbolize the struggle for personal growth, noting that excessive parental involvement in a child’s daily activities can lead to confusion regarding family roles and functions [37, 51]. Furthermore, Lee, Kuo [47] reported that patients often utilize self-starvation as a means to distance themselves from their families. Achieving independence has also become a motivating factor for emerging adults seeking treatment as a means to escape both their illness and parental control [61]. Once patients achieve independence from their parents, episodes of bingeing and purging naturally decline, as pathological eating behaviours are no longer perceived as a viable option [47]. However, navigating their children’s individuality can be a paradoxical challenge for parents [62, 63]. They may strive to foster their children’s independence while expecting compliance and obedience [62]. Adolescents can also experience pressure to remain loyal to family, which challenges their individuality [47, 60].

Individuality is perceived differently among Chinese individuals than among those from Western cultures. de Montgrémier, Moro [48] explored the emotional and financial connections between children and parents of all age groups in China, emphasizing that dependence and independence are not mutually exclusive [40]. Lee, Kuo [47] proposed a distinct form of individuality among Chinese individuals, characterized by a yearning for autonomy while honouring parental authority.

*Theme 3: Family Communication: Conflict Avoidance and Affective Involvement (n = 34)* In qualitative studies, Chinese families emphasize a strong pursuit of harmony [37, 41, 56, 64]. Conflict is often perceived as a negative form of communication [52]. Conflict avoidance and indirect communication are primary coping strategies used by families [41, 52, 57, 64]. Family members prioritize silence over verbal conflicts, underscoring the importance of familial unity and the desire to avoid direct confrontation to preserve relationships [37]. Practices such as self-discipline, indirect expressions of disapproval, saving others’ face, reciprocity, and emphasizing specific relationships are highly valued [37, 41, 57].

Eating symptoms play a role in problem solving within family dynamics. Lee, Kuo [47] and Ma, Capobianco [65] described dieting/purging behaviours as a covert and subtle outlet for young women to release their psychological pain and distress with generational and familial conflicts. These behaviours can help maintain balance between their external and internal conflicts, allowing them to avoid open and direct confrontation [47]. Ma [63] stated that food could serve as a way for children to express conflicts with and discontent and anger towards their parents. Some studies have noted changes in family communication following illnesses. Eating disorders may enhance family communication, and some patients begin expressing divergent opinions to their parents [43, 48, 66]. Despite this, scenes of family conflict are frequently depicted in qualitative descriptions, wherein conflicts between parents and children concerning various aspects of eating disorders commonly emerge [37, 50].

The relationship between family cohesion and eating pathologies lacks definitive conclusions in quantitative studies (*n* = 3). Kok and Tian [67] reported that family cohesion is not related to eating pathology; however, both family cohesion and conflicts are reported to be related to eating pathology in a study by Lee and Lee [68]. Kang [69] also reported that the AN group presented higher levels of family conflicts than did the control group, indicating a greater propensity to openly express anger, aggression, and contradiction.

In qualitative studies, such as Cao [70] noted that participants perceived family communication as superficial and lacking in discussions about emotions. Quantitative studies (*n* = 5) have reported inconclusive findings regarding the relationships between affective involvement and eating pathology. Kok and Tian [67] reported that parental bonding is not significantly associated with the drive for thinness. Conversely, quantitative studies have emphasized the links between parental abuse [71], poor parent‒child relationships [72], intimacy and emotional expression [69], parental rejection [73] and the risk of developing eating disorders and longer illness durations.

*Theme 4: Parental Roles (n = 24)* In the Chinese context, parents have a clear division of labour, with fathers being decision-makers and mothers providing childcare and education [62, 66]. Fathers are perceived as emotionally reserved, serious, and distant, embodying tough love [47, 52]. Mothers are closer to their children [38, 52] and are viewed as empathetic and nurturing [38, 66]. Mothers are also regarded as responsible for meal preparation and caregiving, and they are often blamed after the child falls ill, bearing the greatest share of caregiving burden [37, 39, 51]. In family conflicts, children often align with their mothers, intervening in parental disputes to protect mothers from emotional distress [37, 74]. However, this protective role may have implications for a child’s own emotional well-being.

However, this pattern is inconsistent. Sometimes, the mother is characterized as domineering, overbearing, highly emotional, and immature [47, 65]. Rejecting food is a means of rejecting an individual’s mother [75]. Grandparents and siblings can also disturb parental roles, as children may distance themselves from their parents and align with their grandparents or siblings as a form of opposition [51, 75].

In Tsai, Curbow [58] quantitative study, the potential role of parental roles was recognized through items in the Taiwanese Ethnic Identity Scale such as assess agreement with maternal responsibilities of caring for both in-laws and children; although results for these specific items were not reported separately, it is clear that Taiwanese ethnic identity, inclusive of these family role items, was significantly positively correlated with disordered eating attitudes and behaviours.

*Theme 5: Parental Control (n = 23)* In Chinese families, parents may exert high control over their children’s lives, characterized by overprotection, increased supervision and guidance, and fewer boundaries, which may be linked to eating problems. Quantitative studies (*n* = 3) have yielded conflicting results, with no significant associations among parental control, parental overprotection, and children’s eating issues [58, 67]. Conversely, retrospective overprotection may be linked to disordered eating behaviours in adults [73].

In qualitative studies, Confucianism emphasizes the significant role of parents in controlling and educating children [48, 54]. de Montgrémier, Moro [62] emphasized that parents may perceive their daughters as less capable in making decisions, believing that fathers need to assist them in decision-making. Parents are concerned about and limit their children’s social media exposure [48] and choices regarding their clothing [62], university and education [46, 62], career [46, 62], peer relationships [61] and marriage [46, 62].

Control and power struggles are significant in individuals with eating disorders, in which, by controlling food intake, the child gains a sense of control over their own body and eating [62, 76]. Bulimia also enables patients to control their emotions and increases their sense of self-control [41]. The control exerted by parents over symptoms and treatment—specifically regarding their children’s participation in therapy and their behaviour during treatment—often leads to acts of rebellion [61]. The greater the focus and control from parents, the more likely the child is to resist compliance or exacerbate their symptoms [61].

Academic achievement is the primary focus of parental control. Parents may place high demands on their children’s academic performance, expressing their expectations for their child’s academic success and the sacrifices necessary, and may expect their children to be equally dedicated to their studies [43, 77]. Chan and Ma [78] reported that traditional Chinese parents were less likely to praise their children even after they achieved certain milestones. Academic achievement is deemed even more important than mental health or religious activities [37, 61]. Engagement in church activities or psychological therapy, which is perceived as time-consuming, can adversely affect academic performance [37, 61]. Lagging in academics or not meeting parental expectations is perceived as being rebellious and noncompliant [61], which can lead to anger and criticism [37, 53]. This intense focus on academic success does not allow individuals to prioritize physical well-being and to be aware of their own needs [52]. de Montgrémier, Chen [43] identified academic pressure as a triggering factor for patients, leading to behaviours such as refusing to eat to save time. de Montgrémier, Chen [43] also reported that eating disorders moderate academic pressure and that when a child becomes ill, family members adjust their academic expectations. However, in some cases concerning academic management, contrasting reports have been documented. These cases indicate that parents do not impose high expectations regarding academic performance and instead emphasize health, while the children place significant importance on their studies [61].

*Theme 6: Specific Behaviours (n = 21)*.

1.  *Unhealthy Feeding Practices (n = 14)* Feeding practices are associated with eating issues. In Chinese families, love is often expressed nonverbally [47], with food being a means of symbolizing affection [54]. Children who are overweight are often perceived as receiving adequate parental care and experiencing a sense of happiness [51] and are considered healthy [54]. When children appear too thin, relatives and friends blame their parents for not giving them proper care [51].

Zhang, Chang [79] summarized the traditional feeding habits of Chinese parents, including wanting children to eat more, encouraging overeating, and forced feeding. Patients with eating disorders scored higher in these aspects than healthy controls did [79]. Liu, Cui [73] mentioned that parental pressure to eat demonstrated robust linkages with children’s disordered eating behaviours; however, parental monitoring and restriction of children’s diets were not risk markers for disordered eating behaviours in Chinese women, whereas they were for men [73].

Qualitative studies have reported behaviours related to problematic eating among children. Children may be forced to eat more food to maintain family harmony and avoid conflicts [47, 52]. The consumption of all the food during family meals prepared by the grandmother or mother reflects filial piety [47]. Cheng and Merrick [52] suggested that parents impose feeding schedules on young children, disregarding their natural hunger cues, which hinders their ability to recognize their own physical needs and develop self-soothing and self-regulation skills. With Westernization, aesthetic and traditional feeding practices have caused further complications. Holmes and Ma [80] reported that mothers simultaneously promoted more food intake by adding dishes while exercising caution from overeating.

2) *Negative Family Body Talk (n = 14)* Negative body talk involving critical comments of one’s or others’ bodies, lack of acceptance, and pressure related to body shape and appearance from the family is related to disordered eating behaviours. Among seven quantitative studies addressing this theme, six identified instances of negative family body talk or concerns about weight and appearance, suggesting possible links to eating problems [73, 81–85]. Additionally, Chang, Nie [72] reported that relatives’ dieting behaviours serve as a risk marker for eating disorders. However, specific behaviours—such as parental norms, appearance modelling, and familial pressure to control weight—did not emerge as significant risk markers in two studies [82, 84].

Qualitative studies reported that parents’ attitudes towards eating conveyed distorted messages to their children about food, hunger, and weight [39]. The influence of parental attitudes towards eating on children is more pronounced in China, where family relationships involve high levels of trust and intimacy, than in Western societies [65, 80]. Similarly, Australian adolescents are more influenced by media, whereas in China, the influences of media, peers, and family are relatively balanced according to a quantitative study by Kakar, Fardouly [83]. Notably, in another quantitative study by Chen, Sun [82], teasing by family members was found to play a more significant role in disordered eating behaviours, even though teasing by peers occurred more frequently. Mothers, as primary caregivers, wield a significant influence, as they are more susceptible to gendered appearance judgements and are more conscious of the social repercussions of not adhering to beauty standards [80]. Chan and Ma [39] showed that dieting may be a form of competition between the patient and mother, as both may be engaged in dieting practices. Negative body talk in the family environment can be profoundly harmful and detrimental, potentially serving as a risk factor for the development of eating disorders [80]. Another phenomenon that may occur among Chinese individuals is that patients may be evaluated as overweight, which should serve a trigger for weight loss; however, their families do not perceive this as negative commentary [77]. Instead, they regard it as a form of praise, reflecting the traditional Chinese belief that being overweight is associated with health and prosperity [77].

### Summary

Table 2 gives definitions and examples of the six themes. Table 3 provides quantitative evidence concerning each theme, indicating whether the empirical findings support the corresponding qualitative descriptions. Supplementary Material H provides a summary of each study’s contribution to the identified themes in the meta-synthesis.

**Table 2:** Themes and subthemes with definitions and examples

Theme and subtheme	Definition	*n*	Example of qualitative/quantitative findings
Family value: filial piety	Parental expectations, honor, family hierarchy, and a strong sense of obligation.	2 quanti, 23 quali	*“Growing up in a family like that*,* I had always wanted to be a perfect kid*,* one who would allow my parents to hold their heads up high” * [47]*.* *“For my parents. I don’t want to see them suffer so much pain and I don’t want to be a bu xiao [unfilial] daughter” * [51]*.*
Family value: gender preferences	Gender bias favoring males and sons.	7 quali	The fact that Ya’s parents had no son created a difficult, negative social dynamic for her parents, resulting in their enduring sense of losing face within their extended family – a phenomenon often observed in traditional Chinese contexts. Consequently, Ya felt the full brunt of her parents’ disappointment as she had to fill the gap and shoulder the added responsibilities and demands that came with the absence of a male child [47]. *“We old folks believe that a married daughter is like water being poured out of the front door. The fate of a divorced woman would be sorrowful. Besides*,* her ex-husband is a good man. He beat her because of her bingeing and purging” * [46].
Family task: individuality	Balancing family unity in a collectivist society with the pursuit of individuality.	1 quanti, 21 quali	Due to Chinese culture’s emphasis on collectivist values, the family and interdependence are more important than the individual and independence [52]. Chinese women, different from that of their mothers. According to P6, “*a woman must be strong and independent even though traditionally women are obedient and do not work. It’s not good*” [62].
Family communication: conflict avoidance and affective involvement	Information transmission and reception, affective involvement, family conflict and conflict handling strategies.	6 quanti, 28 quali	AN was a devil that had adversely affected her mood, her relationship with her parents and her study. However, its presence had made her mother more willing to listen to her, which had seldom happened in the past [66]. With her parents, she used the strategy of escaping from direct conflict or communication with them. For instance, she would burst into loud tears like a baby crying for food [64].
Parental roles	Parental roles and divisions.	1 quanti, 23 quali	The division of labour between the parents was rigidly defined, with Mrs Hui assuming a nurturing role in taking care of her husband and child, while the father’s role in childcare was minimal [66].
Parental control	Overprotection, overparenting, and high demanding on children.	4 quanti, 19 quali	She finally agreed to attend the university chosen by her parents, and was unhappy. She gave up her desire for personal achievement and freedom to follow her parents’ advice. It was the triggering factor for her ED [62]. Food choices have become a tool for 17 AN patients (50%) in gaining power in their families and in exerting control over their own lives [51].
Specific behaviours: unhealthy feeding practices	Family meal, food preparation and feeding practices.	2 quanti, 12 quali	In China, parents always force children to eat more food to make sure that their children get enough nutrients for their health, and to satisfy the parents’ psychological need. Chinese parents would become anxious if their children do not eat so much as they have anticipated [79].
Specific behaviours: negative family body talk	Family tease on body image and weight. Family members’ attitudes towards body shape and weight.	7 quanti, 7 quali	A strong association between weight-teasing by family and disordered eating was discovered, whereas weight-teasing by peers was not found to have a significant association with disordered eating [82]. Family weigh‑teasing indirectly leads to disordered eating through psychological distress for native adolescents [81].

Quanti indicates quantitative studies, quali indicates qualitative studies, and an indicates anorexia nervosa. Italics indicate quotes involved in qualitative research

**Table 3:** Summary of quantitative evidence supporting and contradicting each theme

Themes	Positive/supporting results	Negative/unsupported results
Family task: individuality	–	Kang [69] noted compared to the healthy control group, there were no significant differences in independence observed in the AN group. Additionally, no significant differences were found when comparing subtypes classification (AN-R vs. AN-P) or early versus late onset.
Family communication: conflict avoidance and affective involvement	Kang [69] and Lee and Lee [68] noted the conflict was positively correlated with eating pathology. Quantitative studies emphasized the link between parental abuse [71], poor parent-child relationship [72], intimacy and emotional expression [69], parental rejection [73] and the risk of developing eating disorders and longer illness duration.	Kang [69] also noted the AN group exhibited higher levels of conflict compared to the control group, indicating a greater tendency to openly express anger, aggression, and contradiction. Kok and Tian [67] reported that parental bonding is not significantly associated with the drive for thinness.
Parental control	Liu, Cui [73] reported retrospective overprotection may be linked to disordered eating behaviours in adults	Tsai, Curbow [58] and Kok and Tian [67] reported parental control and parental overprotection was not related to eating pathology. Kang [69] reported no significant differences in control between the AN group and healthy control families.
Feeding practices	Zhang, Chang [79] summarized the traditional feeding habits of Chinese parents, including wanting children to eat more, encouraging overeating, and forced feeding. Patients with eating disorders scored higher in these aspects compared to healthy controls [79]. Liu, Cui [73] mentioned that parental pressure to eat demonstrated robust linkages with children’s disordered eating behaviours;	Liu, Cui [73] reported that parental monitoring and restriction of children’s diet were not a risk marker for disordered eating behaviours in Chinese women, while they were for men.
Negative family body talk	Chen, Lin [81]; Chen, Sun [82]; Kakar, Fardouly [83]; Liu, Cui [73]; Liu, Zhang [84] and Yang, Niu [85] noted negative family body talk or concerns about weight and appearance, suggesting possible links to eating problems. Chang, Nie [72] found that relatives’ dieting behaviours served as a risk marker for eating disorders.	Chen, Sun [82] and Liu, Zhang [84] noted specific behaviours—such as parental norms, appearance modeling, and familial pressure to control weight—did not emerge as significant risk markers.

‘Positive/supporting’ reflects results aligned with the qualitative studies, whereas ‘negative/unsupported’ indicates results that contradict or do not corroborate them

AN-R indicates the anorexia nervosa restrictive type, whereas AN-P indicates the anorexia nervosa purging type

Although employed a quantitative research design, Lee and Lee [68] mentioned the concept of filial piety only in the discussion section without quantitative measurement and did not include it in this table

Tsai, Curbow [58] quantitative study incorporated items related to filial piety and family roles within the measurement of Taiwanese ethnic identity. However, the final results were reported using Taiwanese ethnic identity as an overall construct, without specifically examining the relationships between the filial piety and parental role subcomponents and eating pathology. Therefore, these specific findings regarding filial piety and parental role were not presented in this table

## Discussion

On the basis of the family process model, this study systematically reviewed the literature to summarize the vulnerability factors related to eating disorders within Chinese families. After 518 articles were screened, 50 were included. The meta-synthesis identified six themes corresponding to the family process model: [1] family values (e.g., filial piety and gender preferences); [2] family tasks: individuality; [3] family communication: conflict avoidance and affective involvement; [4] parental roles; [5] parental control; and [6] specific behaviours (e.g., unhealthy feeding practices and negative family body talk).

According to the family process model, family values are influenced by culture and subculture [9]. Filial piety frequently appeared in related studies, highlighting its importance in understanding eating disorders within the Chinese cultural context and forming part of the background for discussions of eating pathology. Consistent with the assumptions of the family process model, family values function as an overarching abstract construct that manifests within more specific modules [9]. In the context of this review, filial piety, as a core family value, appears in various dimensions; for instance, patients may adopt conflict-avoidant behaviours by complying with parental expectations out of filial piety. This study integrates findings from different articles regarding the relationship between filial piety and eating pathology; however, the conclusions seem contradictory. In some studies, filial piety appears to contribute to the development of eating problems, whereas in others, it facilitates recovery. This aligns with the dual filial piety model describing the relationship between filial piety and mental health: authoritarian filial piety, which is associated with hierarchy and obedience, is negatively correlated with psychological well-being, whereas reciprocal filial piety, characterized by care and love, is positively correlated with mental health [86, 87]. If filial piety is conceptualized as a hierarchy emphasizing responsibility, sacrifice, or obedience, individuals may use eating pathology as a means of expressing their personal needs, thereby hindering recovery. Conversely, if filial piety is framed as reciprocal care, it may facilitate the recovery process by promoting supportive and mutual relationships. Empirical quantitative evidence supports the association between filial piety—as a component of female gender role socialization—and eating pathology indicators such as dietary restraint and eating concerns among Asian American women [88]. However, more empirical quantitative data is still needed for further explanatory explanations.

Son preferences emerged inductively across 7 of 50 studies (14%), demonstrating persistent cultural significance from 1992 [59] to 2021 [47], despite societal perceptions of gradual improvement [89]. Documentation primarily occurred through single-case descriptions where therapists, observers, or researchers characterized family dynamics. Textual analysis suggests four potential mechanisms linking this bias to eating pathology: First, through resource scarcity dynamics where preferential investment in sons may drive daughters to adopt eating pathology as a way for familial attention [37]. Second, via social comparison processes where daughters’ self-devaluation emerges from contrasting parental treatment toward siblings, potentially generating body dissatisfaction and restrictive behaviours [59]. Third, through intergenerational pressure transmission wherein sonlessness stigma transfers parental shame to daughters, increasing stress that may subsequently elevate vulnerability to disordered eating [47]. Fourth, through daughters being perceived as outsiders - as documented in studies [41, 46, 54] - resulting in deprivation of family belonging and parental misunderstanding/lack of support. Crucially, while quantitative studies firmly establish maternal son preference correlates with adolescent depression [90], parallel empirical validation for eating pathology remains absent. Current evidence consists largely of descriptive observations, demanding targeted mechanistic studies to test these proposed pathways.

In the Western context, individuality supports the ongoing development of the family in family tasks [9]. Minuchin’s psychosomatic family theory explores the challenges of individuation within families affected by anorexia nervosa, where complex dynamics hinder the daughter’s capacity to attain true independence. In this context, a daughter’s symptoms function as mechanisms to disrupt the status quo and challenge pseudoindividuation [91]. This theoretical framework has been employed in numerous studies to elucidate the challenges faced by Chinese patients (e.g., Ma [63]; Ma, Chow [45]). However, in Chinese culture, collectivistic families may influence the pathway through which individuals form identities, an approach towards identity development that departs from that found in individualistic societies [40, 47, 48].

In Chinese culture, concepts such as mianzi (face) and renqing (favour) are commonly utilized as mechanisms for conflict resolution [92]. Compared with Western individuals, Chinese individuals tend to prefer avoidance strategies when managing conflicts, a tendency that is supported by cross-cultural research [93]. The avoidance of conflict is observed in families with eating disorders. Food and eating issues can serve as a means for children to navigate unexpressed conflicts [47, 63, 65] and balance family cohesion and individual needs [47]. However, quantitative studies have indicated that these families experience higher levels of conflict than healthy control groups among Chinese families [69]. It can be inferred that conflict avoidance is not unique to families with eating disorders in China; however, eating pathology may serve as an alternative channel for the expression of underlying relational tensions.

The family process model suggests that changes and interactions in family roles can influence overall family functioning, as role definitions and expectations may differ due to cultural variations [9]. The reviewed articles suggest that traditional Chinese parental roles may foster a mother‒child alliance, which, according to Minuchin’s theory, can lead to an imbalance in family dynamics, as noted in Erriu, Cimino [91]. However, it is important to note that this finding is not absolute and requires further investigation.

The reviewed articles predominantly emphasize parental control over children’s behaviours, a finding corroborated by Sun, Soh [22], which indicates that Asian students exhibit higher levels of maternal overprotection and achievement orientation than their non-Asian peers do. These familial traits are associated with an increased risk of developing eating pathology. Behaviours are characterized by high control; simultaneously, they also function as expressions of love and care [94, 95]. Among Chinese families, food serves as a means of asserting self-control [41, 62, 76]. Eating problems can help reduce stress and emotional tension. The presence of eating disorders can even result in a reduction in these parental expectations [43].

Family-meal interactions represent the external manifestation of rules, beliefs, and behaviours related to dietary practices within the context of family functioning, and this phenomenon has been discussed in both Chinese and Western reviews [12, 91, 96, 97]. In China, encouraging or coercing children to eat more can be a risk factor for disordered eating behaviours, although parental food restrictions are not associated with eating pathology. Conversely, in Western cultures, food restrictions and strict eating rules are risk factors for eating disorders [91], and pressure to eat is not commonly observed. The conflict between traditional feeding practices and Western cultural norms is also reflected within families, where parents encourage their children to eat large quantities (a traditional indicator of health) while simultaneously wishing to prevent them from becoming overweight (a modern concern) [80]. Negative family body talk, which includes critical attitudes towards body shape and appearance, is indeed present in Western reviews [12, 16, 17]. However, peers and siblings, rather than parents, are more commonly the main sources of appearance teasing, with peers having a greater impact in Western countries [13, 17]. In contrast, the influence of parents is more pronounced in collectivist cultures, where familial attitudes towards body image can have a significant impact [65, 80, 82, 83].

In addition to the themes mentioned above, Chan [37] highlighted the impact of the living environment on family functioning, emphasizing that the environment and family dynamics are inseparable. For example, a patient’s limited living space hinders their access to private areas, a factor that is challenging to categorize within any specific theme. Extended families frequently emerge in qualitative descriptions, which do not appear to have explicit counterparts within the family process model; for example, Lee, Kuo [47] highlighted the impact of the relationships between mothers and grandparents. Ma [98] noted that the environmental context in which parents live may influence their understanding of their daughters, which cannot be strictly delineated or compartmentalized.

## Clinical implications

The findings of this review underscore the need for culturally adapted evidence-based treatment models such as family-based therapy for eating disorders within Chinese populations. Consistent with Western paradigms such as the Maudsley model—which emphasize parental involvement as a critical component of recovery rather than attributing blame [99]—our synthesis supports the incorporation of family members as active agents in treatment. Given the strong family orientation prevalent in Chinese culture, interventions may be optimized by engaging respected elders to legitimize therapeutic processes and framing participation as an expression of filial piety to encourage parents to provide care and children to reciprocate through cooperation and respect.

In addition to family-based interventions for eating disorders, individual treatments should also consider the family as a broader contextual factor influencing psychological issues. For example, adolescent-focused therapy for eating disorders [100] emphasizes paying close attention to adolescents’ understanding and expectations of their family relationships. In the context of China, greater emphasis should be placed on exploring and developing culturally appropriate interventions that assist adolescents in navigating family dynamics in a manner that supports the development of their individuality. Concepts such as individuality and filial piety can also be integrated into cognitive therapy to facilitate discussions with patients with eating disorders.

Incorporating feeding practices and addressing negative family body talk represent important preventive strategies in the context of eating disorder prevention, including guidance on moderate feeding, as well as psychoeducation aimed at reducing negative familial discourse related to body shape and weight.

## Limitations

This review relied on an established family process model to maintain coherence but may have overlooked culturally specific themes. Its focus on studies within Chinese populations limits comparisons with Western contexts. The discussion of cultural differences is mainly qualitative and lacks empirical support, reducing its strength. Additionally, the association between family functioning and eating pathology is primarily based on descriptive and meta-synthesized data, which limits causal inferences.

Furthermore, this study encompasses a broad population of Chinese individuals whose experiences vary across ethnicities, political regimes, geographic regions, generations, and levels of acculturation. However, there is insufficient consideration of within-culture heterogeneity within the Chinese population. Future research should aim to incorporate more diverse subpopulations to better capture this complexity and prevent overgeneralization.

Since all the authors of this study are female and the meta-synthesizes numerous studies predominantly led by female participants and female researchers, we acknowledge that the present work may, to some extent, reflect a female perspective. In interpreting concepts related to family roles and gender preferences, it is important to remain cautious of this potential bias.

We recognize that a significant number of included studies are over a decade old. Nonetheless, this review establishes a vital benchmark for research on eating disorders in Chinese families, capturing important cultural themes despite this temporal spread. We emphasize the need for future work to explore recent developments and emerging trends in this rapidly evolving field.

## Supplementary Information

Supplementary material 1.

## Data Availability

No datasets were generated or analysed during the current study.

## Abbreviations

Quanti: Quantitative studies
Quali: Qualitative studies
AN: Anorexia nervosa
BN: Bulimia nervosa
BED: Binge eating disorder
ED: Eating disorder
ANR: Anorexia nervosa restrictive
ANP: Anorexia nervosa purging
