# Supplementary material for: Family functioning and eating disorders in Chinese populations: a systematic review and meta-synthesis
Source: J Eat Disord. 2025 Nov 24;13:269. doi: 10.1186/s40337-025-01453-1 (PMC12641987; doi:10.1186/s40337-025-01453-1)
Supplement: Supplementary file 1 — Supplementary material 1. [file 40337_2025_1453_MOESM1_ESM.docx]

**Supplementary Material A**

**Search terms in PubMed**

("eating disorder*"[Title] OR "eating pathology"[Title] OR anorexia[Title] OR bulimia[Title] OR "binge eating"[Title] OR "disordered eating"[Title]OR"eating problem") AND ("family"[Title]) AND ("systematic review"[Publication Type] OR "systematic review"[Title/Abstract] OR "scoping review"[Title/Abstract]) NOT ("therapy"[Subheading] OR "therapeutics"[MeSH Terms] OR "family therapy"[MeSH Terms] OR "psychotherapy"[MeSH Terms]) NOT ("parental eating disorder*"[Title/Abstract] OR "maternal eating disorder*"[Title/Abstract]) NOT ("obesity"[MeSH Terms] OR "overweight"[MeSH Terms] OR obes*[Title/Abstract] OR overweight[Title/Abstract]) NOT (treatment*[Title] OR intervention*[Title] OR therap*[Title]) AND ("0001/01/01"[Date - Publication] : "2022/12/31"[Date - Publication])

**Search terms in Web of Science**

TI=("eating disorder*" OR "eating pathology" OR anorexia OR bulimia OR "binge eating" OR "disordered eating") AND TI=("family") AND DT=("Review" OR "Systematic Review") NOT TS=("therapy" OR "therapeutics" OR "treatment*" OR "intervention*" OR "therap*") NOT TS=("parental eating disorder*" OR "maternal eating disorder*") NOT TS=("obesity" OR "overweight" OR obes*) (Studies published up to and including December 2022 were included.)

Google Scholar was used as a supplementary database to ensure comprehensive literature coverage and to identify relevant studies not captured through primary search sources.

**Summary of Existing Reviews on Familial factors and Eating Disorders**

| Review | Research question/purpose | Family functioning | Number of included articles | Sample site | Number of participants | Sample type | Gender | Age | Methodology | Meta-analysis | Meta-synthesis | Conclusion |
| --- | --- | --- | --- | --- | --- | --- | --- | --- | --- | --- | --- | --- |
| Dahill et al., 2021 | To explore the relationship between parental appearance-related teasing, specifically concerning weight, shape, or general appearance, and eating problems among adolescents. | Behaviours | 6 | USA 4  Germany 2 | 11650 | Community sample | Mixed gender (4 studies); Only female (2 studies) | 10–19-year-old | Cross-sectional research | No | No | Parental appearance-related teasing significantly impacts adolescents' eating problems, potentially contributing to maladaptive eating behaviours and eating disorders. |
| Godfrey et al., 2013 | To investigate how family mealtime interactions influence disordered eating behaviours during childhood and adolescence. | Behaviours, communication and affective involvement | 12 | Australia 3 Belgium 1 Canada 1 Germany 2 USA 5 | 6916 | Clinical sample | Mixed gender (10 studies); Only female (2 studies) | Adult participants (however, retrospectively examining childhood or adolescent mealtime interactions) | Experimental studies: 2; Observational studies: 10 (including 1 mixed-method study and 9 quantitative studies, comprising 7 cross-sectional and 2 longitudinal studies) | No | No | Negative family mealtime interactions, such as high parental control and family conflict, are positively associated with disordered eating behaviours, while positive interactions may prevent their development. |
| Grogan et al., 2020 | To identify the various non-abuse adverse life experiences encountered by adults diagnosed with eating disorders, with a particular focus on family-related non-abuse adversities | Behaviours, communication and affective involvement | 25 | Australia 1 Austria 1 Canada 1 Germany 1 Italy 4 Netherlands 2 Portugal 3 UK 7 USA 5 | 11680 | Clinical sample | Mixed gender (22 studies); Only female (3 studies) | Adult participants (however, examining adverse events from childhood or adolescence) | Quantitative studies: 20 (including 19 cross-sectional and 1 longitudinal study); Qualitative studies: 4; Mixed-method studies: 1 | No | No | Adults with eating disorders report significantly higher levels of family-related non-abuse adverse life experiences compared to non-clinical samples, with stronger associations between adverse family dynamics and the onset of BN and BED. |
| Hampshire et al., 2022 | To explore the relationship between various parenting styles and disordered eating behaviours among adolescents. | Control and behaviours | 16 | Australia 3 Canada 1 Finland 1 Netherlands 1 Portugal 1 Switzerland 1 UK 3 USA 5 | 25817 | Community sample | Mixed gender (13 studies); Only female (3 studies) | Under 18-year-old | Cross-sectional studies: 8; Longitudinal studies: 8 | No | No | Parenting styles characterized by high control and low responsiveness are indirectly associated with disordered eating symptoms among adolescents, with variations based on the gender of both parents and offspring. |
| Langdon-Daly & Serpell, 2017 | To systematically evaluate the protective factors, present in family systems that may mitigate the risk of young individuals developing eating disorders and disordered eating behaviours. | General family functioning | 10 | UK 1 USA 6 Sweden 1 Australia 1 Spain 1 | 45201 | Community sample | Mixed gender | Any age | Longitudinal studies: 10 | No | No | Supportive family environments and positive familial relationships, particularly regular family meals, significantly reduce the risk of disordered eating behaviours in young individuals. |
| Quiles Marcos et al., 2013 | To investigate the relative contributions of familial pressure perceived by adolescents on the development of eating disorder symptomatology, specifically examining the relationships between dieting behaviours, bulimic symptoms, and body dissatisfaction. | Control and behaviours | 25 | Australia 7 Canada 1 UK 4 USA 10  Netherlands 1 France 1 | 7385  (Pamies et al., 2010 could not be accessed) | Community sample | Only female (13 studies); Only male (2 studies); Mixed gender (9 studies) | Adolescents and emerging adults | Quantitative studies: 23 (including 22 cross-sectional and 1 longitudinal study); Mixed-method studies: 1 | The average effect sizes for family influence on dieting behaviours, body dissatisfaction, and bulimic symptoms were 0.221 (95% CI: 0.120-0.322), 0.224 (95% CI: 0.129-0.320), and 0.225 (95% CI: 0.102-0.348), respectively, with no significant differences observed across genders. This influence is notably lower in comparison to the impact of peers. | No | Family dynamics significantly influence adolescents' eating behaviours and body dissatisfaction, with parental attitudes and weight perceptions playing a critical role in shaping these outcomes, while also acknowledging the need to examine the differential effects across genders. |
| Saltzman & Liechty, 2016 | To systematically review the family correlates of childhood binge eating (C-BE) in children aged 12 and under. | General family functioning | 15 | USA 7 Germany 4 Belgium 4 | 30504 | Community and clinical sample | Only female (6 studies); Mixed gender (9 studies) | Under 12-year-old | Cross-sectional studies: 9; Longitudinal studies: 6 | No | No | Weight-related teasing and parental emotional unresponsiveness are identified as key correlates of C-BE, while parental weight, education level, and socioeconomic status show no significant associations. |

**Supplementary Material B**

**Search Terms among Different Databases**

The search terms used across different databases are as follows. The most recent search was conducted on April 17, 2024, except for the Chinese Social Sciences Citation Index database, which was searched on June 4, 2025.

**SCOPUS (254 articles)**

( TITLE-ABS-KEY ( "eating disorder*" OR anorexia OR bulimia OR "binge eat*" OR "eating symptom*" OR "eating disturbance" OR "disordered eating" ) AND TITLE-ABS-KEY ( chin* OR "hong kong" OR taiwan* ) AND TITLE-ABS-KEY ( famil* ) ) AND PUBYEAR > 1989 AND PUBYEAR < 2024

**EBSCO host (including MEDLINE, APA Psycinfo, APA PsycArticles, Psychology and Behavioral Sciences Collection and etc.)(43 articles, the website automatically removes duplicates, resulting in 35 articles.)**

SU ( "eating disorder*" OR anorexia OR bulimia OR "binge eat*" OR "eating symptom*" OR "eating disturbance" OR "disordered eating" ) AND SU ( Chin* OR "Hong Kong" OR Taiwan* ) AND SU famil*

Academic Search Premier (16)

MEDLINE (12)

APA Psyclnfo (8)

Psychology and Behavioral Sciences Collection (5)

Business Source Premier (1)

ERIC (1)

Search link: https://search.ebscohost.com/login.aspx?direct=true&db=cmedm&db=psyh&db=pdh&db=pbh&db=aph&db=buh&db=eric&db=8gh&db=lxh&db=nfh&db=bwh&db=trh&db=s3h&db=nlebk&db=lfh&db=ufh&db=hev&db=msn&db=ddu&db=e63sww&db=obo&db=e870sww&db=h7i&bquery=SU+(+%26quot%3beating+disorder*%26quot%3b+OR+anorexia+OR+bulimia+OR+%26quot%3bbinge+eat*%26quot%3b+OR+%26quot%3beating+symptom*%26quot%3b+OR+%26quot%3beating+disturbance%26quot%3b+OR+%26quot%3bdisordered+eating%26quot%3b+)+AND+SU+(+Chin*+OR+%26quot%3bHong+Kong%26quot%3b+OR+Taiwan*+)+AND+SU+famil*&cli0=DT1&clv0=199001-202312&lang=zh-cn&type=1&searchMode=Standard&site=ehost-live

**PubMed (73 articles)**

(("eating disorder*"[Title/Abstract] OR anorexia[Title/Abstract] OR bulimia[Title/Abstract] OR "binge eat*"[Title/Abstract] OR "eating symptom*"[Title/Abstract] OR "eating disturbance"[Title/Abstract] OR "disordered eating"[Title/Abstract]) AND (Chin*[Title/Abstract] OR "Hong Kong"[Title/Abstract] OR Taiwan*[Title/Abstract])) AND (famil*[Title/Abstract])

**Web of Science (137 articles)**

((TS=(“eating disorder*” OR anorexia OR bulimia OR “binge eat*” OR “eating symptom*” OR “eating disturbance” OR “disordered eating”)) AND TS=(Chin* OR "Hong Kong" OR Taiwan* )) AND TS=(famil*)

**Chinese Social Sciences Citation Index (CSSCI) (11 articles)**

**Chinese term**

**主题:进食障碍)OR(主题:厌食)OR(主题:贪食)OR(主题:进食紊乱)OR(主题:障碍的进食行为)OR(主题:暴食)AND(主题:家庭)**

(As CSSCI primarily provides Chinese literature, most of which focuses on Chinese populations, we did not impose any regional restrictions during the search process.）

**Supplementary Material C**

**Reference List of Included Articles**

Cao, S., Miao, S., & Tong, J. (2013). A qualitative study on family relationships in patients with eating disorders (in Chinese). *Chinese Journal of Clinical Psychology*, 21(5), 703–707. https://doi.org/10.16128/j.cnki.1005-3611.2013.05.040

Chan, C. Y. Z. (2003). *A discovery process-outcome study: The roles of perceived significant events in the changes of anorexia nervosa patients and their families in family treatment.* The Chinese University of Hong Kong.

Chan, Z. C. Y., & Ma, J. L. C. (2002a). Family themes of food refusal: Disciplining the body and punishing the family. *Health Care for Women International*, *23*(1), 49-58. <https://doi.org/10.1080/073993302753428429>

Chan, Z. C. Y., & Ma, J. L. C. (2002b). Secrets of Self-Starvation. *Journal of International Women's Studies*, *3*(2), 23-37.

Chan, Z. C. Y., & Ma, J. L. C. (2004a). Etiology of anorexia nervosa in Hong Kong: A qualitative social work inquiry. *Child & Family Social Work*, *9*(2), 177-186. [https://doi.org/10.1111/j.1365-2206.2004.00307.x](https://doi.org/https:/doi.org/10.1111/j.1365-2206.2004.00307.x)

Chan, Z. C. Y., & Ma, J. L. C. (2004b). Daughter praising and mother bashing: A case study from Hong Kong. *Health Care for Women International*, *25*(2), 195-207. <https://doi.org/10.1080/07399330490267459>

Chan, Z. C. Y., & Ma, J. L. C. (2006). A feminist family therapy research study: Giving a voice to a girl suffering from anorexia nervosa. *Journal of Feminist Family Therapy: An International Forum*, *17*(2), 41-64. <https://doi.org/10.1300/J086v17n02_03>

Chan, Z. C. Y., & Ma, J. L. C. (2007). Siblings family drama. *Journal of Feminist Family Therapy*, *19*, 25-61. <https://doi.org/10.1300/J086v19n02_02>

Chan, Z. C. Y., & Ma, J. L. C. (2008). Unwrapping the box: dancing with clients. *Health Care for Women International*, *29*(4), 431-445. <https://doi.org/10.1080/07399330701876661>

Chang, W.-w., Nie, M., Kang, Y.-w., He, L.-p., Jin, Y.-l., & Yao, Y.-s. (2015). Subclinical eating disorders among female medical students in Anhui, China: A cross-sectional study. *Nutricion hospitalaria*, *31*(4), 1771-1777.

Chapman, V., & Procopio, M. (1995). A case of anorexia nervosa in a Chinese boy brought up in the United Kingdom. *European Eating Disorders Review, 3(4)*, 255-259. https://doi.org/10.1002/erv.2400030407

Chen, D.-R., Lin, L.-Y., & Levin, B. (2023). Differential pathways of disordered eating among immigrant and native adolescents in Taiwan. *Journal of Eating Disorders*, *11*(1), 54. https://doi.org/10.1186/s40337-023-00781-4

Chen, D.-R., Sun, G., & Levin, B. (2022). Gender-specific responses to multifaceted factors associated with disordered eating among adolescents in 7th to 9th grade. *Journal of Eating Disorders*, *10*(1), 5. https://doi.org/10.1186/s40337-021-00524-3

Chen, G., He, J., Wu, S., Zhang, B., & Fan, X. (2020). Relationship between Overeating and Child Abuse by Family Members among Chinese Adolescents. *Children and Youth Services Review, 112*, 104944. https://doi.org/10.1016/j.childyouth.2020.104944

Cheng, P. H., & Merrick, E. (2016). Cultural adaptation of dialectical behavior therapy for Chinese international students with eating disorders and depression. *Clinical Case Studies, 16*(1), 42-57. <https://doi.org/10.1177/1534650116668269>

de Montgrémier, M. V. A., Chen, J., Zhang, F., & Moro, M. R. (2015). Male anorexia in China: Psychopathological, cultural, and transcultural factors (in French). *L'Évolution Psychiatrique*, *82*(1), 63-74. <https://doi.org/10.1016/j.evopsy.2015.12.004>

de Montgrémier, M. V. A., Lachal, J., Blanchet-Collet, C., & Moro, M. R. (2017). Anorexia nervosa in the daughters of Chinese immigrants: Contributions of transcultural psychiatry (in French). *Neuropsychiatrie de l'Enfance et de l'Adolescence*, *65*(8), 500-507. [https://doi.org/10.1016/j.neurenf.2017.08.004](https://doi.org/https:/doi.org/10.1016/j.neurenf.2017.08.004)

de Montgrémier, M. V. A., Chen, L., Chen, J., Moro, M. R. (2017). Case study of an adopted Chinese woman with bulimia nervosa: A cultural and transcultural approach. *Shanghai Archives of Psychiatry, 29*(4), 243-246. <https://doi.org/10.11919/j.issn.1002-0829.216089>

de Montgrémier, M. V. A., Chen, J., Guo, K., Moro, M. R. (2017). Cultural and transcultural aspects of eating disorders for Chinese female adolescents or young adults (in French) *Neuropsychiatrie de l'Enfance et de l'Adolescence*, *65*(3), 146-154. [https://doi.org/10.1016/j.neurenf.2017.03.002](https://doi.org/https:/doi.org/10.1016/j.neurenf.2017.03.002)

de Montgrémier, M. V. A., Moro, M. R., Chen, J., Blanchet, C., & Lachal, J. (2019). Eating disorders and representation of the role of women in China: A qualitative study. *European Eating Disorders Review 28*(2), 211-222. [https://doi.org/10.1002/erv.2717](https://doi.org/https:/doi.org/10.1002/erv.2717)

de Montgrémier, M. V. A., Moro, M. R., Chen, J., & Lachal, J. (2021). Female patients with eating disorders and their parents experience in China: A qualitative study (in French). *Encephale*, *48*(1), 43-51. <https://doi.org/10.1016/j.encep.2021.01.006> (Vécu de jeunes femmes présentant un trouble des conduites alimentaires et de leurs parents en Chine : étude qualitative.)

Demarque, M., Guzman, G., Morrison, E., Ahovi, J., Moro, M. R., & Blanchet-Collet, C. (2015). Anorexia nervosa in a girl of Chinese origin: Psychological, somatic and transcultural factors. *Clinical Child Psychology and Psychiatry*, *20*(2), 276-288.

Holmes, S., & Ma, H. (2023). A feminist approach to eating disorders in China: A qualitative study. *Journal of Eating Disorders*, *11*(1), 157. https://doi.org/10.1186/s40337-023-00883-z

Kakar, V., Fardouly, J., Rapee, R. M., Guo, M., Arman, S., & Niazi, E. (2023). Exploring the tripartite influence model of body image and disordered eating among adolescent girls living in Australia, China, India, and Iran. *Body Image*, *47*, 101633. https://doi.org/10.1016/j.bodyim.2023.101633

Kang, Q., Chen, J., Jiang, W., Wu, S., Liu, Q., Chen, H., Fan, J., Zhang, H., Zhang, M., & Xiao, Z. (2014). Characteristics of family environment and clinical symptoms in patients with anorexia nervosa (in Chinese). *Chinese Journal of Mental Health, 28(10),* 735–740.

Kok, L., & Tian, C. (1994). Susceptibility of Singapore Chinese schoolgirls to anorexia nervosa-part ii (family factors). *Singapore Medical Journal*, *35*, 609-612.

Lai, K. Y., Pang, A. H., & Wong, C. K. (1995). Case study: Early-onset anorexia nervosa in a Chinese boy. *Journal of the American Academy of Child & Adolescent Psychiatry*, *34*(3), 383-386. https://doi.org/10.1097/00004583-199503000-00025

Lee, A. M., & Lee, S. (1995). Disordered eating and its psychosocial correlates among Chinese adolescent females in Hong Kong. *International Journal of Eating Disorders*, *20*(2), 177-183. https://doi.org/10.1002/(sici)1098-108x(199609)20:2<177::Aid-eat8>3.0.Co;2-d

Lee, S., Hsu, L. K., & Wing, Y. K. (1992). Bulimia nervosa in Hong Kong Chinese patients. *The* *British Journal of Psychiatry*, *161*, 545-551. https://doi.org/10.1192/bjp.161.4.545

Lee, Y., Kuo, B. C. H., Chen, P.-H., & Lai, N.-H. (2021). Recovery from anorexia nervosa in contemporary Taiwan: A multiple-case qualitative investigation from a cultural-contextual perspective [Article]. *Transcultural Psychiatry*, *58*(3), 365-378, Article 1363461520920327. https://doi.org/10.1177/1363461520920327

Liu, K., Zhang, J., Liu, S., Chen, J., Zhang, Y., Li, W., Fu, H., & Fu, L. (2023). Parental stress on children's appearance, body dissatisfaction, and eating Behaviors in Chinese children: A pathway analysis. *Psychological Research and Behavior Management*, *16*, 363-372. https://doi.org/10.2147/prbm.S395628

Liu, Y., Cui, T., Barnhart, W. R., Wang, Q., Yu, Y., & He, J. (2023). Associations among retrospective parenting styles, retrospective food parenting, and current eating behaviors in Chinese adults. *Appetite*, *184*, 106512. https://doi.org/10.1016/j.appet.2023.106512

Luk, S., & Agoha, R. (2014). *The role of socio-cultural factors in the course of anorexia nervosa: A case of anorexia nervosa in a Chinese-American adolescent*. Taylor & Francis. https://doi.org/10.1080/17542863.2013.783090

Ma, J. L. C. (2005a). The diagnostic and therapeutic uses of family conflicts in a Chinese context: The case of anorexia nervosa. *Journal of Family Therapy*, *27*(1), 24-42. [https://doi.org/10.1111/j.1467-6427.2005.00297.x](https://doi.org/https:/doi.org/10.1111/j.1467-6427.2005.00297.x)

Ma, J. L. C. (2005b). Family treatment for a Chinese family with an adolescent suffering from anorexia nervosa: A case study. *The Family Journal*, *13*(1), 19-26. <https://doi.org/10.1177/1066480704269178>

Ma, J. L. C. (2006). Living in poverty: A qualitative inquiry of emaciated adolescents and young women coming from low-income families in a Chinese context. *Child & Family Social Work*, *12*(2), 152-160. <https://doi.org/10.1111/j.1365-2206.2006.00453.x>

Ma, J. L. C. (2007). Meanings of eating disorders discerned from family treatment and its implications for family education: The case of Shenzhen. *Child & Family Social Work*, *12*(4), 409-416. [https://doi.org/10.1111/j.1365-2206.2007.00496.x](https://doi.org/https:/doi.org/10.1111/j.1365-2206.2007.00496.x)

Ma, J. L. C. (2008). Eating disorders, parent–child conflicts, and family therapy in Shenzhen, China. *Qualitative Health Research*, *18*(6), 803-810.

Ma, J. L. C. (2014). The father's roles in the recovery of young Chinese suffering from anorexia nervosa in two Chinese cities, Hong Kong and Shenzhen. *Journal of Family Therapy*, *37*(1), 61-78. [https://doi.org/10.1111/1467-6427.12037](https://doi.org/https:/doi.org/10.1111/1467-6427.12037)

Ma, J. L. C., & Chan, Z. C. Y. (2004). The different meanings of food in Chinese patients suffering from anorexia nervosa: Implications for clinical social work practice. *Social Work in Mental Health*, *2*(1), 47-70. <https://doi.org/10.1300/J200v02n01_04>

Ma, J. L. C., Chow, M. Y. M., Lee, S., & Lai, K. (2002). Family meaning of self‐starvation: Themes discerned in family treatment in Hong Kong. *Journal of Family Therapy*, *24*(1), 57-71. <https://doi.org/10.1111/1467-6427.00201>

Ma, J. L. C., & Lai, K. (2007). Perceived treatment effectiveness of family therapy for chinese patients suffering from anorexia nervosa: A qualitative inquiry. *Journal of Family Social Work*, 10(2), 59-74. https://doi.org/10.1300/J039v10n02_04

Ma, R. F., Capobianco, K. P., Buchanan, N. T., Hu, Z., & Oakman, J. M. (2019). Etiologic and treatment conceptualizations of disordered eating symptoms among mainland Chinese therapists. *International Journal of Eating Disorders*, *53*(3), 391-403. [https://doi.org/10.1002/eat.23204](https://doi.org/https:/doi.org/10.1002/eat.23204)

Ma, R. F., Zhang, M. N., Oakman, J. M., Wang, J. P., Zhu, S. Q., Zhao, C. D., Hu, Z. Y., & Buchanan, N. T. (2021). Eating disorders treatment experiences and social support: Perspectives from service seekers in mainland China. *International Journal of Eating Disorders*, *54*(8), 1537-1548. <https://doi.org/10.1002/eat.23565>

Schmidt, U. (1993). Bulimia nervosa in the Chinese. *International Journal of Eating Disorders*, *14*(4), 505-509. https://doi.org/10.1002/1098-108X(199312)14:4<505::AID-EAT2260140415>3.0.CO;2-N

Tong, J., Miao, S. J., Wang, J., Zhang, J. J., Wu, H. M., Li, T., & Hsu, L. K. (2005). Five cases of male eating disorders in central China. *International Journal of Eating Disorders*, *37*(1), 72-75. https://doi.org/10.1002/eat.20061

Tsai, G., Curbow, B., & Heinberg, L. (2003). Sociocultural and developmental influences on body dissatisfaction and disordered eating attitudes and behaviors of Asian women. *The Journal of Nervous and Mental Disease*, *191*(5), 309-318. https://doi.org/10.1097/01.Nmd.0000066153.64331.10

Xu, W. (2007). *Factors and processes influencing motivation for psychotherapy of anorexia nervosa sufferers in mainland China: A qualitative research in case of Shenzhen (in Chinese).* The Chinese University of Hong Kong.

Yang, W., Niu, G., Shi, X., Song, K., Zhang, Y., & Yuan, Z. (2023). Negative family body talk and negative emotional eating among Chinese adolescent girls and young adult women: The role of body dissatisfaction and feminism consciousness. *Appetite*, *188*, 106973. https://doi.org/10.1016/j.appet.2023.106973

Zhang, R., Chang, G., Li, L., & Zhang, Z. (2015). The effects of Chinese traditional feeding habits on the eating disorders and the psychological analysis. *Eating behaviors*, *17*, 83-85. https://doi.org/10.1016/j.eatbeh.2015.01.003

**Supplementary Material D**

**Summary of Qualitative or Case Studies**

| Authors/year | Language | Age  M(range/SD)^1^ | Sex male/female (female percentage)^2^ | Samples | Location | Diagnosis | Theory | Data collection | Analysis | Main theme^3^ |
| --- | --- | --- | --- | --- | --- | --- | --- | --- | --- | --- |
| Cao et al., 2013 | Chinese | 22-29 | 0/23(100%) | 23 patients | Mainland | 9 BN  5 AN  9 BED | Culture in eating pathology  Family therapy theory | Depth interview | Thematic analysis | - Family relationships - Communication |
| Chan & Ma, 2002a | English | 40 | 0/1(100%) | 1 patient | Hong Kong | AN | Family therapy theory | Videotaping the family therapy sessions | Content analysis | - Filial Piety - Conflict avoidance |
| Chan & Ma, 2002b | English | 24 | 0/1(100%) | 1 patient | Hong Kong | AN | Family therapy theory/socio-cultural perspective | Interview | Matching and thematization | - Filial piety - Family relationship - Competence with mother on dieting |
| Chan & Ma, 2004a | English | 17.25(15-24) | 0/8(100%) | 8 patients | Hong Kong | 6 AN, 2 BN | Biological-psychological-social theory | Multiple source data collection method | Coding-thematization | - Family relationships |
| Chan & Ma, 2004b | English | CT | 0/1(100%) | 1 patient | Hong Kong | AN | Transcultural theory | Clinical case | Comments to the clinical case | - Lacking maternal praise |
| Chan & Ma, 2006 | English | CT | 0/1(100%) | 1 patient | Hong Kong | AN | Feminist epistemology and a client-driven approach | The patient selected specific family therapy session recordings for review, engaging in a process of examination and interpretation to elucidate her experiences with anorexia nervosa, her personal background, and the therapeutic interventions. | Analyzing and categorizing comments made by the female patient in the family therapy session recordings. | - Parent-child relationships - Sibling relationships - Family conflicts |
| Chan & Ma, 2007 | English | 21 | 0/1(100%) | 1 patient | Hong Kong | AN | Various theories in the field of family therapy include externalization theory, narrative therapy, and postmodernist approaches. | Interviews, observations, and documentation of patients and family members. | Qualitative research methods involve integrating interview content and observational findings to reveal changes in patient symptoms and the dynamics of family relationships. | - Mother-child relationship - Father-child relationship - Sibling relationship |
| Chan & Ma, 2008 | English | 14 | 0/1(100%) | 1 patient | Hong Kong | AN | Gender perspective/family dynamics | Treatment interview | NA | - Family relationships - Family role - Conflict avoidance |
| Chan, 2003 | English | 19(16-23) | 0/4(100%) | 4 patients and their families | Hong Kong | AN | Systemic family therapy  with a client-driven approach | Multiple case of family interviews, post-treatment interviews | Analysis videotapes of family treatment sessions | - Family relationships - Family conflict |
| Chapman & Procopio, 1995 | English | 15 | 1/0(0%) | 1 patient | UK | AN | Cross-cultural adaptation theory | Interviews, observations, and documentation of patients and family members. | NA | - Parenting - Conflicts between traditional family values and the western |
| Cheng & Merrick, 2016 | English | 24 | 0/1(100%) | 1 patient | USA | AN-P | Dialectical behaviour therapy | Treatment interview | NA | - Filial piety - Family value - Family relationships |
| de Montgrémier et al., 2015 | French | 23 | 1/0(0%) | 1 patient | Mainland | AN | Transcultural psychology | Clinical case record | NA | - Family relationships - Tensions between individual and family expectation |
| de Montgrémier, Lachal, et al., 2017 | French | CT | 0/1(100%) | 1 patient | France | AN | Cross-culture psychiatry | Clinical observation and qualitative interview | Case analysis | - Family dynamics in migration |
| de Montgrémier, Chen, Chen, et al., 2017 | English | 23 | 0/1(100%) | 1 patient | France | BN | Transcultural psychiatry and psychoanalysis and complementarism | Interviews and observations | Case analysis | - Individuality |
| de Montgrémier, Chen, Guo, et al., 2017 | French | 17.125(13-23) | 0/8（100%） | 8 patients | France | 6 AN  2 BN | Transcultural psychiatry | Clinical cases | Theme analysis | - Generation gap - Parenting - Conflicts between personal goals and parental expectations |
| de Montgrémier et al., 2019 | English | 19.9(13-31) | 0/11(100%) | 11 patients and 8 parents | Mainland | 7 AN  4 BN | Transcultural psychiatry | Semi-structured interview | Interpretative phenomenological analysis | - Family labor division - Women's status in the family - Parental expectations for daughters - Intergenerational conflicts within the family |
| de Montgremier et al., 2021 | French | 19.3(12-31) | 0/13(100%) | 13 patients and 11 patients’ parents | Mainland | 9 AN, 4 BN | Interpretative phenomenological analysis | Semi-structured interview | Identify themes and meta-themes by reading the interview over and over again | - Generation conflict - Parenting |
| Demarque et al., 2015 | English | 18 | 0/1(100%) | 1 patient | France | AN | Cross-cultural psychological analysis | Clinical case record and family interview | NA | - Family dynamics in migration - Family relationships |
| Holmes & Ma, 2023 | English | 25(20-29) | 0/12(100%) | 12 patients | Mainland | 6 AN  4 BN  2 EDNOS | Feminist approaches | Interview | Reflexive thematic analysis | - Family meals - Family members' comments and expectations |
| Lai et al., 1995 | English | 11 | 1/0(0%) | 1 patient | Hong Kong | 1 AN | Multifactorial model of the etiology of anorexia and family dynamics | Clinical case record | NA | - Favoring sons over daughters - Family relationships - Family communication |
| Lee et al., 1992 | English | 4 | 0/4(100%) | 4 patient | Hong Kong | 4 BN | Biopsychosocial theory | Case report | NA | - Favoring sons over daughters |
| Lee et al., 2021 | English | CT | 0/3(100%) | 3 patients | Taiwan | 1 AN-R, 1 AN-P, 1 EDNOS | Acculturation perspective/social constructivist philosophy | In-depth semi-structured interviews and follow-up interviews | An inductive (data-driven) and deductive (theory-driven) approach to topic analysis | - Filial piety - Individuality - Family meals - Conflict avoidance |
| Luk & Agoha， 2014 | English | 15 | 0/1(100%) | 1 patient | America | AN | Transcultural theory and feminist theory | A retrospective chart review of the single medical record | Case analysis | - Family meals - Parental control - Individuality |
| Ma & Chan, 2004 | English | 15.7 (12 - 25) | 0/34(100%) | 34 patients and their families | Hong Kong | AN | Sociocultural theory and family systems theory | Interview | Thematization | - Family relationships - Filial piety |
| Ma et al., 2002 | English | 17.8(14-24) | 0/5(100%) | 5 patients and their families | Hong Kong | AN | Family systems theory | Recorded dialogue of family therapy session | Content analysis | - Coalition of daughter with mother - Family loyalty |
| Ma et al., 2019 | English | 44.56(10.29) | 9/32(78%) | 41 psychotherapists | Mainland | NA | Psychoanalytic or Psychodynamics | In-depth, semi-structured telephone interviews | Directed content analysis | - Family relationships - Role of mothers - Family cohesion |
| Ma et al., 2021 | English | CT | CT | 31 patients | Mainland | AN | CT | Semi-structured interviews. Interview data collection methods include telephone interview and written form | Inductive data-driven thematic analysis with deductive coding | - Guilt and filial piety - Past generations' experiences of famine and cultural expectations |
| Ma, 2005a | English | 14.4(10-22) | CT | 35 patients and their families | Hong Kong | AN | Family systems theory | Family interviews | Content analysis | - Conflict strategy - Family relationships |
| Ma, 2005b | English | 15 | 0/1(100%) | 1 patient | Hong Kong | AN | Family therapy theory | Methods such as semi-structured interviews, observational records, and family meeting notes. | NA | - Family conflicts - Filial piety |
| Ma, 2006 | English | 15.7(14-21) | 0/7(100%) | 7 patients and their families | Hong Kong | AN | Structural family therapy | In-depth pre-treatment and post-treatment interviews, and video recordings of family therapy sessions | Content analysis to identify key topics and patterns | - Family relationships - Filial piety |
| Ma, 2007 | English | 15.7(14-21) | 0/7(100%) | 7 patients from low-income families | Hong Kong | AN | Socio-cultural theory, family systems theory, attachment theory and social capital theory | Intensive interviews at the pre-treatment and post-treatment phases | Thematization | - Parental control - Family relationships |
| Ma, 2008 | English | CT | 0/10(100%) | 10 patients and their families | Mainland | 5 AN, 5 BN | Family system theory | Therapeutic video (recording), in-depth interviews and follow-up interviews | Content analysis | - Family conflict - Filial piety |
| Ma, 2014 | English | 16.5(15-18) | 0/2(100%) | 2 patients and their families | Mainland & Hong Kong | 2 AN | Family system theory | Clinical case record | NA | - Family conflict - Family labor division |
| Ma & Lai, 2007 | English | Adolescents:14 (1.59) at referral; Adults: 23.4 (7.7) | 0/24(100%) | 24 patients and their families | Hong Kong | AN | Social constructionism | Post-treatment interviews | Thematization | - Parental control |
| Schmidt, 1993 | English | 22-30 | 1/2(67%) | 3 patients | England | BN | Cultural transition | Clinical case record | NA | - Family environment - Family value - Family dynamics in migration |
| Tong et al., 2005 | English | 18.8(15-23) | 5/0(0%) | 5 patients | Mainland | 2 AN-R.2 AN-P,1 BN | CT | Clinical observation, interview, psychological evaluation and investigation of social and cultural background of the cases | Case analysis | - Family environment - Parental relationship |
| Xu, 2007 | Chinese | 19.9(13-25) | 0/10(100%) | 10 Patients | Mainland | AN | Trans-Theoretical Model | Therapeutic video (recording), in-depth interviews and follow-up interviews | Text analysis | - Family relationships - Family interactions |

Note: AN, anorexia nervosa; AN-P, anorexia nervosa–purging subtype; AN-R, anorexia nervosa–restrictive subtype; BN, bulimia nervosa; EDNOS, eating disorders not otherwise specified; NA, not applicable; CT, cannot tell. 1. In this study, when interviews were conducted with both patients and their family members, only the patient's age was considered. 2. When interviews were conducted with both patients and their family members, only the patient's sex ratio was calculated. 3. Only themes related to the family and disease onset are listed in this section.

Ma et al. (2021) incorporated a quantitative screening component; however, the study primarily focused on semi-structured qualitative interviews, emphasizing an in-depth exploration and analysis of qualitative data. Accordingly, in this review, we classify it within the qualitative research category.

**Supplementary Material E**

**Summary of Quantitative Studies**

| Authors/year | Language | Age range/M(SD) | Sex Male/Female (Female percentage) | Samples | Location | Independent variable | Dependent variable | Coefficient | Qualitizing^1^ |
| --- | --- | --- | --- | --- | --- | --- | --- | --- | --- |
| Chang et al., 2015 | English | 17-25 | 0/1107(100%) | 1107 | Mainland, Wuhan | - Relationship with parents - Parent’s relationship - Dieting status among relatives | - Risk of developing eating disorders | - β_relationship with parents_=0.118, p<0.001 - β_dieting status among relatives_=-0.083，p<0.01 - Parents’ relationship was not included in the regression model due to a lack of statistical significance | Poor relationship with parents and dieting behaviours of relatives were identified as factors contributing to an increased risk of eating disorders. |
| Chen et al., 2020 | English | 14.46(1.38) | 2110/2236(51.4%) | 4346 | Mainland, | Child abuse | - Overeat and binge eating | - Abused by father   OR_boy_=1.49, p<0.05  OR_girl_=1.78, p<0.05  Abused by mother  OR_boy_=1.13, p>0.05  OR_girl_=1.37, p<0.05   - Abused by other family members   OR_boy_=1.86, p<0.05  OR_girl_=1.54, p>0.05 | Boys and girls who reported higher levels of child abuse perpetrated by  their father, mother, and other family members showed higher levels of overeating. |
| Chen et al., 2022 | English | 13.71(0.93) | 357/372(51.03%) | 729 | Taiwan | - Weight talk within the family - Pressure from the family to control weight - Perceived family support | - Disordered eating | - OR_family weight-teasing_=1.79, p<0.001 - OR_family pressure to control weight_=1.23, p=0.47 - OR_perceived family support_=1.01, p=0.93 | Family weight-teasing may be associated with disordered eating in adolescents. |
| Chen et al., 2023 | English | 13.83(0.98) | 357/372(51.03%) | 729 | Taiwan | - Family weight talk | - Disordered eating | r=0.30, AOR=1.35, p=0.33 | Family weight talk is not directly associated with disordered eating; however, there is an indirect relationship mediated by depressive mood. |
| Kakar et al., 2023 | English | 14.02(1.15) | 0/293(100%) | 293 | Mainland | Family pressures (including the expectation or influence of family members on appearance, weight, image, etc.) | - Disordered eating | r=0.19, p<0.001 | Family pressures are significantly associated with disordered eating behaviours among the Chinese population. Chinese individuals reported similar levels of pressure from family, peers, and media. |
| Kang et al., 2014 | Chinese | 19(3) | 0/93(100%) | 93 | Mainland | - Family environment | - Eating attitudes - Duration of Illness | Eating attitudes  r_Intimacy_ = -0.24, p < 0.05  r_Conflict_ = 0.32, p < 0.05  Duration of illness  r_Intimacy_ = -0.27, p < 0.05  r_Emotional Expression_ = -0.28, p < 0.05  r_Entertainment_ = -0.23, p < 0.05 | Family environment of AN patients is characterized by low intimacy, emotional expression, and entertainment, alongside high conflict, with low intimacy linked to more severe psychopathology, worse eating attitudes, and longer illness duration. |
| Kok & Tian, 1994 | English | 16(CT) | 0/656(100%) | 656 | Singapore | - Family adaptability - Family cohesion - Parental bonding | - Drive for thinness | There were no significant differences between the high drive for thinness and low drive for thinness groups in terms of family functioning, family cohesion, family adaptability, parents’ overprotection and care. | The high drive and low drive for thinness group showed no significant differences in family factors. There were also no significant differences in overprotection and caregiving between these two groups. Therefore, family factors may not be a significant etiological factor for anorexia nervosa. |
| Lee & Lee, 1995 | English | 15.87(1.46) | 0/294(100%) | 294 | Hong Kong | - Family cohesion - Family conflict | - Disordered eating | - β_perceived family cohesion_=0.178, p<0.01 - β_perceived family conflict_=0.117, p<0.05 | Perceived family cohesion and conflict both exhibit positive predictive relationships with disordered eating behaviours. Excessive family cohesion may present challenges for some adolescents in their pursuit of autonomy. |
| Liu, Cui, et al., 2023 | English | 19-46 | 251/250(49.7%) | 501 community sample | Mainland | - Retrospective parenting styles (e.g., emotional warmth, rejection, and overprotection) - Food socialization (e.g., parental concern, monitoring, pressure to eat, and restriction) | - Disordered eating | - r_retrospective warmth_=-0.007(women)/0.001(men),p>0.05 - r_retrospective rejection_=0.26(women)/0.27(men),p<0.001 - r_retrospective overprotection_=0.34(women)/0.23(men),p<0.001 - r_retrospective concern_=0.24(women)/0.33(men),p<0.001 - r_retrospective monitoring_=0.10, p>0.05(women)/0.13(men) p<0.05 - r_retrospective pressure to eat_=0.17, p<0.01(women)/0.16, p<0.05(men) - r_retrospective restriction_=0.12, p>0.05(women)/0.20, p<0.01(men) | Retrospective parenting styles and food socialization are significantly associated with current maladaptive eating behaviors. |
| Liu Zhang, et al., 2023 | English | Boys  11.37 (1.76)  Girls  11.54(1.80) | 315/284(47.39%) | 599 students | Mainland | The pressure from parents on children's appearance (including parental pestering, unfairness and ignorance, encouragement, and normative shaping behavior) | - Unhealthy eating behaviors | r_parental teasing score_=0.13(boy),p<0.01/0.18,p<0.05(girl)  r_parental injustice and ignorance score_=0.09, p>0.05(boy)/0.08, p<0.05(girl)  r_parental encouragement score_=0.08(boy)/0.09(girl), p>0.05  r_parental norms and modelling score_=0.09(boy)/0.11(girl), p>0.05 | In Chinese children and adolescents, there is an association between parental pressure on children's appearance and children's body dissatisfaction and eating behaviors. |
| Tsai et al., 2003 | English | Taiwanese-American subjects  19.4(3.2)  Taiwanese subjects  18.1(1.7) | 0/645(100%) | 298 Taiwanese-American subjects, 347 Taiwanese subjects | Taiwan | Parental bonding, the Taiwanese ethnic identity (Scale includes items related to filial piety, family roles, and parental control.) | - Disordered eating attitudes and behaviors | Taiwanese-American subjects  β_maternal overprotection_=0.06, p>0.05  β_paternal overprotection_=0.05, p>0.05  β_Taiwanese ethnic identity_=0.20, p<0.001  Taiwanese subjects  β_maternal overprotection_=0.04, p>0.05  β_paternal overprotection_=-0.01, p>0.05  β_Taiwanese ethnic identity_=0.18, p<0.001 | The study did not support the association between parental control and the development of eating disorders. However, the Taiwanese e  thnic identity which includes items regarding filial piety and family roles, have been found to be significantly associated with disordered eating behaviors and attitudes. |
| Yang et al., 2023 | English | 19.4(1.25) | 0/813(100%) | 813 college students | Mainland | Negative body talk | - Negative emotional eating | Negative emotional eating  r=0.17, p<0.01  Body dissatisfaction  r=0.26, p<0.01 | Negative body talk within the family is positively correlated with emotional eating behaviors and body dissatisfaction. |
| Zhang et al., 2015 | English | Patients  17.6(2.58) | CT | 35 patients and 35 normal female youths, along with their parents | Mainland | Traditional feeding practices (including the expectation for children to consume larger quantities of food, encouragement of overeating, and in some cases, the use of force feeding) | - Presence of eating issues | t_(patient vs control)_= -4.97(youth assessment)/-3.62(parents assessment), p<0.001 | Adolescents with eating issues are more likely to recall their parents' traditional feeding practices. |

Note: OR, odds ratio; AOR, adjusted odds ratio. 1. A process in which quantitative findings were translated into textual descriptions and themes to facilitate integration with qualitative data.

**Supplementary Material F**

**Quality Assessment of Qualitative or Case Studies**

| Criteria | 1 | 2 | 3 | 4 | 5 | 6 | 7 | 8 | 9 | 10 | Rating |
| --- | --- | --- | --- | --- | --- | --- | --- | --- | --- | --- | --- |
| Cao et al., 2013 | Yes | Yes | Yes | Yes | Yes | Yes | Yes | Yes | Yes | Very Valuable | Good |
| Chan & Ma, 2002a | Yes | Yes | No | Yes | Yes | Yes | Yes | Yes | Yes | Valuable | Fair |
| Chan & Ma, 2002b | Yes | Yes | No | Yes | No | Yes | Yes | Yes | Yes | Valuable | Fair |
| Chan & Ma, 2004a | Yes | Yes | Yes | Yes | Yes | Yes | Yes | Yes | Yes | Very Valuable | Good |
| Chan & Ma, 2004b | Yes | Yes | No | No | No | Yes | Yes | Yes | Yes | Valuable | Fair |
| Chan & Ma, 2006 | Yes | Yes | No | Yes | Yes | Yes | Yes | Yes | Yes | Valuable | Fair |
| Chan & Ma, 2007 | Yes | Yes | No | Yes | Yes | Yes | Yes | Yes | Yes | Valuable | Fair |
| Chan & Ma, 2008 | Yes | Yes | No | Yes | No | Yes | Yes | Yes | Yes | Valuable | Fair |
| Chan, 2003 | Yes | Yes | Yes | Yes | Yes | Yes | Yes | Yes | Yes | Valuable | Fair |
| Chapman & Procopio, 1995 | Yes | Yes | No | Yes | No | Yes | Yes | Yes | Yes | Valuable | Fair |
| Cheng & Merrick, 2016 | Yes | Yes | No | Yes | No | Yes | Yes | Yes | Yes | Valuable | Fair |
| de Montgrémier et al., 2015 | Yes | Yes | No | No | No | Yes | Yes | Yes | Yes | Valuable | Fair |
| de Montgrémier, Lachal, et al., 2017 | Yes | Yes | No | Yes | Yes | Yes | Yes | Yes | Yes | Valuable | Fair |
| de Montgrémier, Chen, Chen, et al., 2017 | Yes | Yes | No | Yes | No | Yes | Yes | Yes | Yes | Valuable | Fair |
| de Montgrémier, Chen, Guo, et al., 2017 | Yes | Yes | Yes | No | Yes | Yes | Yes | Yes | Yes | Very Valuable | Good |
| de Montgrémier et al., 2019 | Yes | Yes | Yes | Yes | Yes | Yes | Yes | Yes | Yes | Very Valuable | Good |
| de Montgrémier et al., 2021 | Yes | Yes | Yes | Yes | Yes | Yes | Yes | Yes | Yes | Very Valuable | Good |
| Demarque et al., 2015 | Yes | Yes | No | Yes | No | Yes | Yes | Yes | Yes | Valuable | Fair |
| Holmes & Ma, 2023 | Yes | Yes | Yes | Yes | Yes | Yes | Yes | Yes | Yes | Very Valuable | Good |
| Lai et al., 1995 | Yes | Yes | No | No | No | Yes | Yes | Yes | Yes | Valuable | Fair |
| Lee et al., 1992 | Yes | Yes | No | No | No | Yes | Yes | Yes | Yes | Valuable | Fair |
| Lee et al., 2021 | Yes | Yes | No | Yes | Yes | Yes | Yes | Yes | Yes | Valuable | Fair |
| Luk & Agoha, 2014 | Yes | Yes | No | No | Yes | Yes | Yes | Yes | Yes | Valuable | Fair |
| Ma & Chan, 2004 | Yes | Yes | Yes | Yes | Yes | Yes | Yes | Yes | Yes | Very Valuable | Good |
| Ma et al., 2002 | Yes | Yes | Yes | Yes | Yes | Yes | Yes | Yes | Yes | Valuable | Fair |
| Ma et al., 2019 | Yes | Yes | Yes | Yes | Yes | Yes | Yes | Yes | Yes | Very Valuable | Good |
| Ma et al., 2021 | Yes | Yes | Yes | Yes | Yes | Yes | Yes | Yes | Yes | Very Valuable | Good |
| Ma, 2005a | Yes | Yes | Yes | Yes | Yes | Yes | Yes | Yes | Yes | Very Valuable | Good |
| Ma, 2005b | Yes | Yes | Yes | Yes | No | Yes | Yes | Yes | Yes | Valuable | Fair |
| Ma, 2006 | Yes | Yes | Yes | Yes | Yes | Yes | Yes | Yes | Yes | Very Valuable | Good |
| Ma, 2007 | Yes | Yes | Yes | Yes | Yes | Yes | Yes | Yes | Yes | Very Valuable | Good |
| Ma, 2008 | Yes | Yes | Yes | Yes | Yes | Yes | Yes | Yes | Yes | Very Valuable | Good |
| Ma, 2014 | Yes | Yes | No | No | No | Yes | Yes | Yes | Yes | Valuable | Fair |
| Ma & Lai, 2007 | Yes | Yes | Yes | Yes | Yes | Yes | Yes | Yes | Yes | Very Valuable | Good |
| Schmidt, 1993 | Yes | No | No | No | No | No | Yes | Yes | Yes | Valuable | Fair |
| Tong et al., 2005 | Yes | Yes | No | Yes | Yes | Yes | Yes | Yes | Yes | Valuable | Fair |
| Xu, 2007 | Yes | Yes | Yes | Yes | Yes | Yes | Yes | Yes | Yes | Very Valuable | Good |

Note: 1. Is the research purpose clear and well defined? 2. Are these research methods reasonable? 3. Are study participants appropriate? 4. Is the data-collection method appropriate? 5. Is the data analysis method appropriate? 6. Are the results presented adequately? 7. Is the interpretation of the results reasonable? 8. Are the conclusions based on the research results? 9. Is there a clear statement of findings? 10. Is this qualitative study valuable?

Given their inherently descriptive and exploratory nature that aligns with qualitative research methodologies, case reports were also appraised using this checklist.

**Supplementary Material G**

**Quality Assessment of Quantitative Studies**

| Criteria | 1 | 2 | 3^1^ | 4 | 5 | 8 | 9^2^ | 11^2^ | 12 | 14 | Rating |
| --- | --- | --- | --- | --- | --- | --- | --- | --- | --- | --- | --- |
| Chang et al., 2015 | Yes | Yes | Yes | Yes | No | Yes | No | NR | NA | Yes | Fair |
| Chen et al., 2020 | Yes | Yes | Yes | Yes | No | Yes | NR | NR | NA | Yes | Fair |
| Chen et al., 2022 | Yes | Yes | Yes | Yes | Yes | Yes | Yes | Yes | NA | Yes | Good |
| Chen et al., 2023 | Yes | Yes | Yes | Yes | No | Yes | No | NR | NA | Yes | Fair |
| Kakar et al., 2023 | Yes | Yes | NR | Yes | No | Yes | NR | NR | NA | Yes | Fair |
| Kang et al., 2014 | Yes | Yes | NR | Yes | No | Yes | NR | NR | NA | Yes | Fair |
| Kok & Tian, 1994 | Yes | Yes | Yes | Yes | No | Yes | NR | NR | NA | Yes | Fair |
| Lee & Lee, 1995 | Yes | Yes | Yes | Yes | No | Yes | NR | NR | NA | Yes | Fair |
| Liu, Cui, et al., 2023 | Yes | Yes | NR | Yes | No | Yes | Yes | Yes | NA | Yes | Good |
| Liu Zhang, et al., 2023 | Yes | Yes | NR | Yes | Yes | Yes | Yes | No | NA | Yes | Good |
| Tsai et al., 2003 | Yes | Yes | Yes | Yes | No | Yes | Yes | Yes | NA | Yes | Good |
| Yang et al., 2023 | Yes | Yes | Yes | Yes | No | Yes | Yes | Yes | NA | Yes | Good |
| Zhang et al., 2015 | Yes | Yes | NR | Yes | No | Yes | No | Yes | NA | Yes | Fair |

Note: NA, not applicable; NR, not reported

The criteria for the quality assessment were as follows: 1. Was the research question or objective clearly stated? 2. Was the study population clearly specified and defined? 3. Was the participation rate at least 50%? 4. Were all the participants selected or recruited from the same or similar populations (including the same time)? Were inclusion and exclusion criteria for the study prespecified and applied uniformly to all participants? 5. Was a sample size justification, power description, or variance and effect estimates provided? 6. For the analyses in this paper, were the exposure(s) of interest measured prior to the outcome(s) being measured? 7. Was the timeframe sufficient so that one could reasonably expect to see an association between exposure and outcome if it existed? 8. For exposures that can vary in amount or level, did the study examine different levels of the exposure as related to the outcome (e.g., categories of exposure, or exposure measured as a continuous variable)? 9. Were the exposure measures (independent variables) clearly defined, valid, reliable, and implemented consistently across all study participants? 10. Was the exposure(s) assessed more than once over time? 11. Were the outcome measures (dependent variables) clearly defined, valid, reliable, and implemented consistently across all study participants? 12. Were the outcome assessors blinded to the exposure status of participants? 13. Was the loss to follow-up after baseline 20% or less? 14. Were key potential confounding variables measured and adjusted statistically for their impact on the relationship between exposure(s) and outcome(s)?

Considering that items 6, 7, 10, and 13 are primarily designed to assess the quality of longitudinal studies, and given that none of the included studies employed a longitudinal design, these items were not evaluated in the present review.

1. The ratio of invited participants to completed or valid responses ≥ 50% is considered as meeting the requirements for item 3.
2. The measurement of both exposure and outcome variables was evaluated for reliability by referencing Cronbach’s alpha coefficients to assess internal consistency. Some studies only reported the scales used without providing the Cronbach's alpha coefficients for those scales within their own samples, and thus were classified as NR in items 9 and 11. Cronbach's alpha coefficients below 0.7 were classified as "no" for items 9 and 11. Additionally, the use of self-developed scales or the simple yes-or-no question regarding some aspect were also categorized as "no" for items 9 and 11.

**Supplementary Material H**

**Summary of Each Study’s Contribution to the Meta-synthesis Themes**

| Author, year | Location | Family values | | Family tasks: Individuality | Family communication | Family role | Parental control | Specific behaviours | |
| --- | --- | --- | --- | --- | --- | --- | --- | --- | --- |
|  |  | Filial piety | Gender preferences |  | : Conflict avoidance and affective involvement |  |  | Feeding practices | Negative family body talk |
| Cao et al., 2013 | Mainland | - | - | ✓ | ✓ | - | ✓ | - | - |
| Chan & Ma, 2002a | Hong Kong | ✓ | - | - | ✓ | - | - | - | - |
| Chan & Ma, 2002b | Hong Kong | ✓ | - | - | ✓ | ✓ | - | ✓ | ✓ |
| Chan & Ma, 2004a | Hong Kong | ✓ | - | - | ✓ | - | - | - | - |
| Chan & Ma, 2004b | Hong Kong | - | - | - | - | - | ✓ | - | - |
| Chan & Ma, 2006 | Hong Kong | - | - | ✓ | - | - | - | - | - |
| Chan & Ma, 2007 | Hong Kong | - | - | - | - | ✓ | - | - | - |
| Chan & Ma, 2008 | Hong Kong | - | - | - | ✓ | ✓ | ✓ | - | - |
| Chan, 2003 | Hong Kong | ✓ | ✓ | ✓ | ✓ | ✓ | ✓ | ✓ | ✓ |
| Chang et al., 2015 | Mainland | - | - | - | ✓ | - | - | - | ✓ |
| Chapman & Procopio, 1995 | UK | - | - | - | - | - | ✓ | - | ✓ |
| Chen et al., 2020 | Mainland | - | - | - | ✓ | - | - | - | - |
| Chen et al., 2022 | Taiwan | - | - | - | - | - | - | - | ✓ |
| Chen et al., 2023 | Taiwan | - | - | - | - | - | - | - | ✓ |
| Cheng & Merrick, 2016 | Taiwan | ✓ | - | ✓ | ✓ | ✓ | ✓ | ✓ | ✓ |
| de Montgrémier et al., 2015 | Mainland | ✓ | - | ✓ | ✓ | ✓ | ✓ | - | - |
| de Montgrémier, Lachal, et al., 2017 | France | ✓ | ✓ | - | ✓ | - | ✓ | ✓ | - |
| de Montgrémier, Chen, Chen, et al., 2017 | Mainland | ✓ | ✓ | ✓ | ✓ | - | ✓ | - | - |
| de Montgrémier, Chen, Guo, et al., 2017 | Mainland | ✓ | - | ✓ | ✓ | - | ✓ | - | - |
| de Montgrémier et al., 2019 | Mainland | ✓ | - | ✓ | ✓ | ✓ | ✓ | - | - |
| de Montgrémier et al., 2021 | Mainland | ✓ | - | ✓ | ✓ | - | ✓ | - | - |
| Demarque et al., 2015 | France | - | - | ✓ | ✓ | ✓ | - | ✓ | - |
| Holmes & Ma, 2023 | Mainland | ✓ | - | ✓ | - | - | - | ✓ | ✓ |
| Kakar et al., 2023 | Mainland | - | - | - | - | - | - | - | ✓ |
| Kang et al., 2014 | Mainland | - | - | ✓ | ✓ | - | ✓ | - | - |
| Kok & Tian, 1994 | Singapore | - | - | - | ✓ | - | ✓ | - | - |
| Lai et al., 1995 | Hong Kong | ✓ | ✓ | ✓ | ✓ | - | ✓ | - | - |
| Lee et al., 1992 | Hong Kong | - | ✓ | - | ✓ | ✓ | - | - | - |
| Lee & Lee, 1995 | Hong Kong | ✓ | - | - | ✓ | - | - | - | - |
| Lee et al., 2021 | Taiwan | ✓ | ✓ | ✓ | ✓ | ✓ | - | ✓ | - |
| Liu, Cui, et al., 2023 | Mainland | - | - | - | ✓ | - | ✓ | ✓ | ✓ |
| Liu, Zhang, et al., 2023 | Mainland | - | - | - | - | - | - | - | ✓ |
| Luk & Agoha， 2014 | US | - | - | ✓ | - | - | - | - | - |
| Ma & Chan, 2004 | Hong Kong | ✓ | - | ✓ | ✓ | ✓ | ✓ | ✓ | - |
| Ma et al., 2002 | Hong Kong | ✓ | - | ✓ | ✓ | ✓ | ✓ | - | - |
| Ma et al., 2019 | Mainland | - | - | ✓ | ✓ | ✓ | - | ✓ | ✓ |
| Ma et al., 2021 | Mainland | ✓ | - | - | - | ✓ | - | - | - |
| Ma, 2005a | Hong Kong | ✓ | - | - | ✓ | ✓ | - | ✓ | - |
| Ma, 2005b | Hong Kong | ✓ | - | ✓ | ✓ | ✓ | - | - | - |
| Ma, 2006 | Hong Kong | ✓ | - | - | ✓ | ✓ | - | - | - |
| Ma, 2007 | Mainland | - | - | - | ✓ | ✓ | ✓ | - | - |
| Ma, 2008 | Mainland | ✓ | ✓ | ✓ | - | ✓ | ✓ | - | - |
| Ma, 2014 | Mainland & Hong Kong | - | - | - | ✓ | ✓ | - | - | - |
| Ma & Lai, 2007 | Hong Kong | - | - | - | - | - | ✓ | - | - |
| Schmidt, 1993 | UK | ✓ | - | ✓ | ✓ | ✓ | - | ✓ | ✓ |
| Tong et al., 2005 | Mainland | - | - | - | ✓ | ✓ | - | - | - |
| Tsai et al., 2003 | Taiwan & US | ✓ | - | - | - | ✓ | ✓ | - | - |
| Xu, 2007 | Mainland | ✓ | - | ✓ | ✓ | ✓ | ✓ | ✓ | - |
| Yang et al., 2023 | Mainland | - | - | - | - | - | - | - | ✓ |
| Zhang et al., 2015 | Mainland | - | - | - | - | - | - | ✓ | - |
|  |  | 25 | 7 | 22 | 34 | 24 | 23 | 14 | 14 |

Note: ✓ includes a description or discussion about the theme. – does not include a description or discussion of the theme. Quanti means quantitative study, while quali means qualitative study.

**Supplementary Material I**

PRISMA 2020 Checklist

| **Section and Topic** | **Item #** | **Checklist item** | **Location where item is reported** |
| --- | --- | --- | --- |
| **TITLE** | | |  |
| Title | 1 | Identify the report as a systematic review. | 1 |
| **ABSTRACT** | | |  |
| Abstract | 2 | See the PRISMA 2020 for Abstracts checklist. | 1 |
| **INTRODUCTION** | | |  |
| Rationale | 3 | Describe the rationale for the review in the context of existing knowledge. | 2-4 |
| Objectives | 4 | Provide an explicit statement of the objective(s) or question(s) the review addresses. | 4 |
| **METHODS** | | |  |
| Eligibility criteria | 5 | Specify the inclusion and exclusion criteria for the review and how studies were grouped for the syntheses. | 5-7 |
| Information sources | 6 | Specify all databases, registers, websites, organisations, reference lists and other sources searched or consulted to identify studies. Specify the date when each source was last searched or consulted. | 5 & Declarations & Supplementary Material B |
| Search strategy | 7 | Present the full search strategies for all databases, registers and websites, including any filters and limits used. | Supplementary Material B & Declarations |
| Selection process | 8 | Specify the methods used to decide whether a study met the inclusion criteria of the review, including how many reviewers screened each record and each report retrieved, whether they worked independently, and if applicable, details of automation tools used in the process. | 7 |
| Data collection process | 9 | Specify the methods used to collect data from reports, including how many reviewers collected data from each report, whether they worked independently, any processes for obtaining or confirming data from study investigators, and if applicable, details of automation tools used in the process. | 7 |
| Data items | 10a | List and define all outcomes for which data were sought. Specify whether all results that were compatible with each outcome domain in each study were sought (e.g. for all measures, time points, analyses), and if not, the methods used to decide which results to collect. | Supplementary Material D & E |
|  | 10b | List and define all other variables for which data were sought (e.g. participant and intervention characteristics, funding sources). Describe any assumptions made about any missing or unclear information. | Supplementary Material D & E |
| Study risk of bias assessment | 11 | Specify the methods used to assess risk of bias in the included studies, including details of the tool(s) used, how many reviewers assessed each study and whether they worked independently, and if applicable, details of automation tools used in the process. | 7 |
| Effect measures | 12 | Specify for each outcome the effect measure(s) (e.g. risk ratio, mean difference) used in the synthesis or presentation of results. | NA |
| Synthesis methods | 13a | Describe the processes used to decide which studies were eligible for each synthesis (e.g. tabulating the study intervention characteristics and comparing against the planned groups for each synthesis (item #5)). | 7-8 |
|  | 13b | Describe any methods required to prepare the data for presentation or synthesis, such as handling of missing summary statistics, or data conversions. | 7-8 |
|  | 13c | Describe any methods used to tabulate or visually display results of individual studies and syntheses. | 7-8 |
|  | 13d | Describe any methods used to synthesize results and provide a rationale for the choice(s). If meta-analysis was performed, describe the model(s), method(s) to identify the presence and extent of statistical heterogeneity, and software package(s) used. | 7-8 |
|  | 13e | Describe any methods used to explore possible causes of heterogeneity among study results (e.g. subgroup analysis, meta-regression). | NA |
|  | 13f | Describe any sensitivity analyses conducted to assess robustness of the synthesized results. | NA |
| Reporting bias assessment | 14 | Describe any methods used to assess risk of bias due to missing results in a synthesis (arising from reporting biases). | NA |
| Certainty assessment | 15 | Describe any methods used to assess certainty (or confidence) in the body of evidence for an outcome. | NA |
| **RESULTS** | | |  |
| Study selection | 16a | Describe the results of the search and selection process, from the number of records identified in the search to the number of studies included in the review, ideally using a flow diagram. | 8-9 |
|  | 16b | Cite studies that might appear to meet the inclusion criteria, but which were excluded, and explain why they were excluded. | 6 |
| Study characteristics | 17 | Cite each included study and present its characteristics. | Supplementary Material C & D & E |
| Risk of bias in studies | 18 | Present assessments of risk of bias for each included study. | NR |
| Results of individual studies | 19 | For all outcomes, present, for each study: (a) summary statistics for each group (where appropriate) and (b) an effect estimate and its precision (e.g. confidence/credible interval), ideally using structured tables or plots. | NA |
| Results of syntheses | 20a | For each synthesis, briefly summarise the characteristics and risk of bias among contributing studies. | NR |
|  | 20b | Present results of all statistical syntheses conducted. If meta-analysis was done, present for each the summary estimate and its precision (e.g. confidence/credible interval) and measures of statistical heterogeneity. If comparing groups, describe the direction of the effect. | NA |
|  | 20c | Present results of all investigations of possible causes of heterogeneity among study results. | NA |
|  | 20d | Present results of all sensitivity analyses conducted to assess the robustness of the synthesized results. | NA |
| Reporting biases | 21 | Present assessments of risk of bias due to missing results (arising from reporting biases) for each synthesis assessed. | NA |
| Certainty of evidence | 22 | Present assessments of certainty (or confidence) in the body of evidence for each outcome assessed. | NA |
| **DISCUSSION** | | |  |
| Discussion | 23a | Provide a general interpretation of the results in the context of other evidence. | 19 |
|  | 23b | Discuss any limitations of the evidence included in the review. | 20 |
|  | 23c | Discuss any limitations of the review processes used. | 20 |
|  | 23d | Discuss implications of the results for practice, policy, and future research. | 19-20 |
| **OTHER INFORMATION** | | |  |
| Registration and protocol | 24a | Provide registration information for the review, including register name and registration number, or state that the review was not registered. | Declarations |
|  | 24b | Indicate where the review protocol can be accessed, or state that a protocol was not prepared. | Declarations |
|  | 24c | Describe and explain any amendments to information provided at registration or in the protocol. | Declarations |
| Support | 25 | Describe sources of financial or non-financial support for the review, and the role of the funders or sponsors in the review. | Declarations |
| Competing interests | 26 | Declare any competing interests of review authors. | Declarations |
| Availability of data, code and other materials | 27 | Report which of the following are publicly available and where they can be found: template data collection forms; data extracted from included studies; data used for all analyses; analytic code; any other materials used in the review. | Declarations |

Note: NA not applicable; NR not reported. PRISMA means Preferred Reporting Items for Systematic reviews and Meta-Analyses.
